# Baicalein attenuates extracellular matrix remodeling via FN1 in diabetic kidney disease: a multi-omics, machine-learning and mesangial-cell rescue study

**DOI:** 10.3389/fphar.2026.1852694

**Published:** 2026-09-09

**Authors:** Yimin Li, Yuqing Tian, Zhenhua Wu, Fangyu Xia, Liyifan Zheng, Meifang Ren, Meijiao Zhao, Qian Zhang, Jinchuan Tan, Qingyou Xu, Fengwen Yang

**Affiliations:** 1 Department of Nephrology, Hebei University of Traditional Chinese Medicine, Shijiazhuang, China; 2 The First Department of Nephrology, Hebei Provincial Hospital of Traditional Chinese Medicine, Shijiazhuang, China

**Keywords:** baicalein, diabetic kidney disease, extracellular matrix remodeling, FN1, machine learning, single-cell RNA sequencing

## Abstract

**Methods:**

Three transcriptomic cohorts, GSE30528, GSE96804, and GSE104948, comprising 62 DKD and 59 control samples, were integrated for differential expression and weighted gene co-expression network analyses. Candidate genes were prioritized using disease databases, protein–protein interaction analysis, 113 machine-learning algorithm combinations, and SHAP analysis. Model performance was evaluated in GSE30529 and GSE99339. The prioritized genes were then subjected to reverse network pharmacology, followed by FN1 validation in GSE47184 and surface plasmon resonance (SPR). An independently generated mouse single-cell RNA-sequencing dataset comprising 36,819 cells from three DKD and three control mice was subsequently used to localize Fn1 expression. Finally, db/db mouse experiments and an Fn1-overexpression rescue experiment in high-glucose-treated mesangial cells were performed.

**Results:**

We identified 441 differentially expressed genes, including 197 upregulated and 244 downregulated genes. Intersection analysis yielded 94 candidate genes. The blue WGCNA module contained 1,346 genes and was positively correlated with DKD (r = 0.59, P = 1 × 10–12). The glmBoost model achieved the highest mean area under the receiver operating characteristic curve (0.938), and SHAP analysis prioritized FN1, COL1A2, VCAN, FBN1, and COL4A1. Reverse network pharmacology mapped baicalein only to FN1. GSE47184 independently confirmed the direction of FN1 upregulation in DKD. SPR detected a concentration-dependent interaction between baicalein and immobilized recombinant human FN1, with an apparent KD of 8.04 × 10–5 M. Single-cell analysis localized Fn1 expression to mesangial cells, and subsequent mouse-level pseudobulk analysis showed increased mesangial Fn1 expression in DKD mice. Baicalein reduced serum creatinine and blood urea nitrogen levels, ameliorated renal histopathological injury, and decreased fibronectin and other fibrosis-associated molecules in db/db mice. In mesangial cells, Fn1 overexpression aggravated the high-glucose-induced profibrotic molecular phenotype and partially attenuated the inhibitory effects of baicalein on ECM-associated molecular changes.

**Conclusion:**

Baicalein attenuated renal injury and ECM remodeling in experimental DKD. FN1 was functionally involved in the profibrotic phenotype and the cellular response to baicalein.

## 1 Introduction

Diabetic kidney disease (DKD) is one of the most common and severe microvascular complications of diabetes (Alicic et al., 2017). With the continued rise in global diabetes prevalence, DKD has become one of the leading causes of end-stage renal disease (ESRD) (Zhang et al., 2025). Current clinical interventions primarily rely on renin–angiotensin–aldosterone system (RAAS) inhibitors including angiotensin-converting enzyme inhibitors and angiotensin receptor blockers, and sodium–glucose cotransporter 2 (SGLT2) inhibitors. These medications exert renoprotective effects by reducing intraglomerular pressure and through mechanisms that are partly independent of glycemic and blood-pressure control. In addition, nonsteroidal mineralocorticoid receptor antagonists (nsMRAs) have also been shown to further delay disease progression through antifibrotic and anti-fibrotic mechanisms (Bakris et al., 2020). However, current treatment strategies remain unable to completely halt the progression of DKD, particularly in patients with moderate-to-advanced disease, who remain at increased risk of progressive renal function decline and kidney failure (Su et al., 2025). Therefore, additional therapeutic strategies targeting the molecular processes that drive progressive kidney injury are needed.

DKD is driven by interacting metabolic, hemodynamic, and cellular disturbances rather than by a single signaling pathway or renal cell type (Hu et al., 2024). Under conditions of sustained metabolic stress, phenotypic alterations in intrinsic renal cells and excessive accumulation of extracellular matrix (ECM) components promote progressive renal fibrosis (Gao et al., 2025). High-throughput transcriptomic and single-cell RNA-sequencing technologies provide important tools for elucidating the cellular composition and molecular characteristics of DKD (Tan et al., 2024). Fibronectin, encoded by the *FN1* gene, is an important ECM component involved in matrix organization and cell–matrix signaling. However, computational prioritization alone cannot determine whether increased *FN1* expression merely reflects ECM accumulation or contributes functionally to the profibrotic phenotype.

Baicalein is a major flavonoid compound isolated from the roots of the traditional Chinese medicinal plant *Scutellaria baicalensis* Georgi (Munjal et al., 2024). It has attracted widespread attention due to its broad biological activity, particularly its anti-inflammatory, antioxidant, and tissue-protective effects (Zhao et al., 2024). Baicalein can alleviate inflammatory responses by regulating inflammation-related signaling pathways such as NF-κB and MAPK (Yang X. et al., 2025), while also exerting tissue-protective effects by inhibiting oxidative stress. Furthermore, some studies suggest that baicalein has been reported to modulate TGF-β1-related signaling pathways (Sahin et al., 2025), thereby inhibiting extracellular matrix deposition and reducing tissue fibrosis. However, he molecular processes associated with the effects of baicalein in DKD, particularly its relationship with *FN1*-associated ECM remodeling, remain incompletely understood.

**FIGURE 1:**
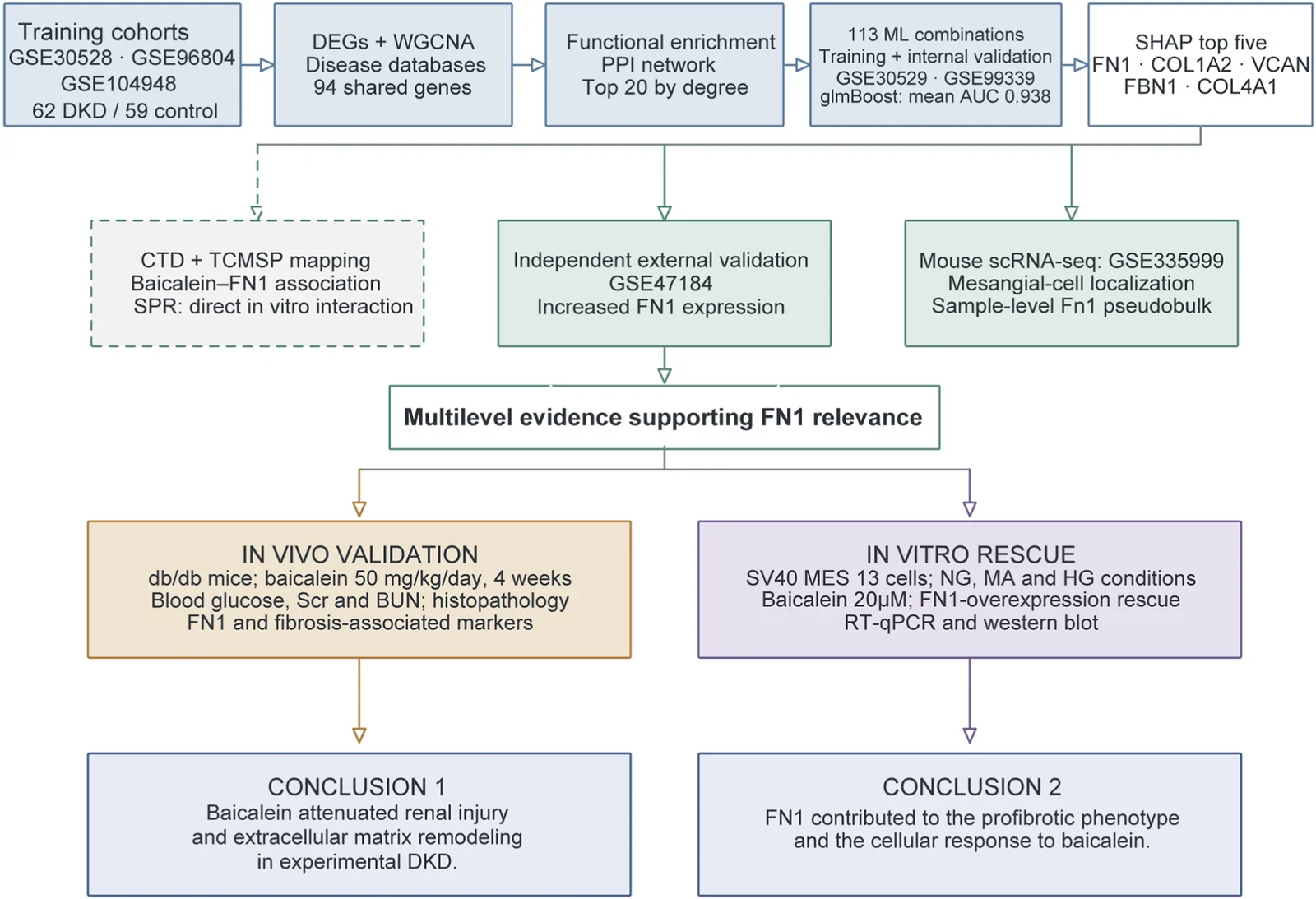
Study workflow and evidence framework. Three transcriptomic training cohorts (GSE30528, GSE96804, and GSE104948), comprising 62 diabetic kidney disease (DKD) and 59 control samples, were integrated for differential expression analysis and weighted gene co-expression network analysis (WGCNA). Intersection with disease-associated genes yielded 94 shared genes, which were subjected to functional enrichment and protein–protein interaction (PPI) analyses. The 20 genes with the highest degree centrality were entered into 113 machine-learning combinations. Model performance was evaluated in the training set and two internal validation cohorts (GSE30529 and GSE99339). The glmBoost model achieved the highest mean area under the receiver operating characteristic curve (AUC = 0.938), and Shapley additive explanations (SHAP) analysis prioritized FN1, COL1A2, VCAN, FBN1, and COL4A1. CTD- and TCMSP-based mapping identified a database-reported association between baicalein and FN1. Surface plasmon resonance (SPR) subsequently detected a concentration-dependent interaction between baicalein and immobilized recombinant human FN1, with an apparent *K*D of 8.04 × 10^−5^ M (80.4 μM). The dashed arrow denotes the branch initiated by database association mapping. The SPR result supports a direct *in vitro* interaction. Increased FN1 expression was independently validated in GSE47184. Mouse single-cell RNA-sequencing data from GSE335999 first localized *Fn1* expression to mesangial cells, and subsequent mouse-level pseudobulk analysis showed increased mesangial *Fn1* expression in DKD mice. This multilevel evidence supported subsequent experimental evaluation. *In vivo*, db/db mice received baicalein at 50 mg/kg/day for 4 weeks, followed by assessments of blood glucose, serum creatinine (SCr), blood urea nitrogen (BUN), renal histopathology, *Fn1*/fibronectin, and fibrosis-associated markers. *In vitro*, SV40 MES 13 cells were examined under normal-glucose, mannitol osmotic-control, and high-glucose conditions. Baicalein treatment and an FN1-overexpression rescue design were followed by RT-qPCR and Western blot analyses. The combined evidence indicated that baicalein attenuated renal injury and extracellular matrix remodeling in experimental DKD and that FN1 contributed to the profibrotic phenotype and the cellular response to baicalein. Solid arrows indicate the analytical, validation, and experimental sequence.

In this study, we integrated multi-cohort glomerular transcriptomic data with weighted gene co-expression network analysis, machine learning, and SHAP-based feature-contribution ranking to prioritize DKD-associated candidate genes. Model performance was assessed in two validation cohorts, while FN1 expression was independently evaluated in GSE47184. Reverse network pharmacology and surface plasmon resonance (SPR) were used to examine the database-derived association and direct *in vitro* interaction between baicalein and FN1. Independently generated mouse single-cell RNA-sequencing data were used to localize *Fn1* expression and evaluate its disease-associated alteration at the cellular level. The effects of baicalein on renal functional indices, histopathological injury, and ECM-associated molecular changes were examined in db/db mice. Finally, an FN1-overexpression rescue experiment was performed in high-glucose-treated mesangial cells to determine whether increased FN1 expression aggravates the profibrotic molecular phenotype and attenuates the cellular effects of baicalein. This design distinguishes database-derived associations, direct *in vitro* interaction, and functional cellular involvement, without assuming cellular or *in vivo* target engagement. The overall study design is illustrated in Figure 1.

## 2 Materials and methods

### 2.1 Data acquisition and preprocessing

The National Center for Biotechnology Information (NCBI) GEO database (http://www.ncbi.nlm.nih.gov/geo) contains a wide variety of high-throughput functional genomics data (Cox et al., 2025). This study included three transcriptomic datasets related to DKD: GSE30528 (GPL571, 9 cases of DKD, 13 normal kidney tissue samples), GSE96804 (GPL17586, 41 cases of DKD, 20 normal kidney tissue samples), and GSE104948 (GPL22945 and GPL24120, 12 cases of DKD, 26 normal kidney tissue samples). Batch effects were corrected for the merged expression matrix using the ComBat method from the sva package in R (Yu et al., 2024). The correction efficacy was evaluated via box plots and principal component analysis (PCA) (Supplementary Figure S1). Ultimately, the three datasets were integrated to obtain a total of 62 DKD samples and 59 normal kidney tissue samples as the training set for differential expression analysis and machine-learning model construction. GSE30529 (10 DKD cases and 12 normal renal tissue samples) and GSE99339 (14 DKD cases and 8 normal renal tissue samples) were used as internal validation sets to assess model stability and identify key genes. GSE47184 (18 DKD cases and 7 normal renal tissue samples) served as an external validation set to further validate the model’s accuracy and the reliability of the identified key genes. Single-cell RNA-sequencing data were generated in-house from kidney samples of 3 DKD (db/db) and 3 control (db/m) mice and deposited in the GEO database under accession number GSE335999.

### 2.2 Differential expression analysis (DEGs)

Based on the batch-effect–corrected expression matrix, differentially expressed genes (DEGs) between DKD and normal control samples were identified using the limma package in R. Background correction and normalization were performed with the normexp and normalizeBetweenArrays functions. Genes with |log_2_ fold change| > 0.585 (corresponding to a fold change of 1.5) and an FDR-adjusted *P* value (adj.P.Val) < 0.05 were considered differentially expressed. Heatmaps and volcano plots were generated using the pheatmap and ggplot2 packages.

### 2.3 Weighted gene co-expression network analysis (WGCNA)

WGCNA is used to identify highly correlated gene modules (Langfelder and Horvath, 2008). In this study, the “WGCNA” package in R was used for analysis. Samples were subjected to 20 rounds of hierarchical clustering to remove outliers. The pickSoftThreshold function was employed to evaluate the degree of scale-free topological fit for different soft threshold exponents (β) (Zhu et al., 2021). Taking both R^2^ and average connectivity into account, we set β = 8 to construct a scale-free network and calculate the topological overlap matrix (TOM). We then assessed the correlations between module eigengenes and DKD status. Modules with an absolute correlation coefficient of |r| > 0.5 and *P* < 0.05 were considered DKD-associated candidate modules.

### 2.4 Integration of DKD-related disease targets

Using the keyword “diabetic kidney disease,” we searched the GeneCards database (https://www.genecards.org), OMIM (https://www.omim.org), and TTD (https://ttd.idrblab.cn) for DKD-associated genes (Stelzer et al., 2016; Amberger et al., 2019; Wang et al., 2020). The analysis included GeneCards targets with a relevance score of ≥10. Genes retrieved from the three databases were merged and deduplicated to generate a DKD-associated gene set. This gene set was then intersected with the differentially expressed genes and genes belonging to the DKD-associated WGCNA modules. Genes shared among the three gene sets were defined as DKD-associated candidate genes. All databases were accessed on 6 June 2026.

### 2.5 Functional enrichment analysis

We employed Kyoto Encyclopedia of Genes and Genomes (KEGG) pathway analysis (Yu et al., 2012) and Gene Ontology (GO) functional annotation (Ashburner et al., 2000). Terms with *P* < 0.05 were considered statistically significant. Significantly enriched GO terms were visualized using a circular plot and a bar plot, whereas significantly enriched KEGG pathways were presented using a Sankey diagram and a bar plot.

### 2.6 Construction of a protein-protein interaction network

Using the STRING database (https://string-db.org/; accessed 6 June 2026), the DKD-associated candidate genes identified by intersection analysis were submitted (Szklarczyk et al., 2019), The organism was set to *Homo sapiens*, the minimum required interaction score was set to 0.700 (high confidence). And disconnected nodes were hidden. The resulting network was imported into Cytoscape version 3.10.4 for visualization and topological analysis. Degree centrality was calculated, and the top 20 nodes ranked by degree were selected for subsequent analyses.

### 2.7 Construction of 113 machine learning model combinations and feature contribution analysis

This study established a machine-learning evaluation framework covering 12 machine learning algorithms, including regularized regression methods (Lasso, Ridge, Elastic Net), generalized linear model extensions (StepGLM, GLMBoost, PLS-GLM), ensemble learning algorithms (Random Forest, GBM, XGBoost), and classical classifiers (SVM, LDA, and Naive Bayes). Subsequently, a dual-algorithm cascade architecture was employed: first, algorithms with feature selection capabilities were used to filter high-dimensional expression data; second, the resulting feature sets were input into different classifiers to build predictive models. Through systematic algorithm combinations, a total of 113 model permutations were generated (e.g., “Lasso + XGBoost” or “Random Forest + SVM”), and models with fewer than 5 features were excluded to avoid retaining models based on an insufficient number of features (Li T. et al., 2025). All models were evaluated using the integrated training set and two internal validation cohorts, GSE30529 and GSE99339, with their discriminatory performance measured by the area under the receiver operating characteristic curve (AUC). The mean AUC across the three cohorts was used to rank the models. The model with the highest mean AUC was selected, and its retained features were defined as candidate genes for subsequent analyses.

To quantify the contribution of individual features to model predictions, this study employed the SHapley Additive Explanations (SHAP) method to perform feature contribution analysis on the model with the best predictive performance (Dong et al., 2025). The mean absolute SHAP value of each gene was calculated to rank its contribution to distinguishing DKD from control samples. Genes with higher contributions were prioritized as candidate genes for subsequent experimental validation. SHAP-based rankings were interpreted as measures of feature contribution rather than evidence of causality or direct drug-target relationships.

### 2.8 Diagnostic models and roc curve analysis

The feature contributions within the best-performing machine-learning model were quantified using the SHAP (SHapley Additive exPlanations) method, and Genes were ranked by their mean absolute SHAP values, and the top five were retained as candidate genes. To evaluate the diagnostic capability of the candidate genes, ROC curves were generated to distinguish between DKD and control samples (Li N. et al., 2025), and the AUC and its 95% confidence interval (CI) were calculated. Based on previous studies, an AUC > 0.75 was considered to indicate relatively strong discriminatory performance (Dong et al., 2025).

### 2.9 Single-cell RNAsequencing analysis

The single-cell RNA sequencing data were generated from renal tissues of three db/db mice with DKD and three db/m control mice, and have been deposited in the Gene Expression Omnibus (GEO) under accession number GSE335999.

Raw 10× Genomics H5 count matrices were imported into R and processed with Seurat (v5.4.0) (Stuart et al., 2019). Seurat objects were created with a minimum of three cells per gene and 200 detected genes per cell. Cells with 300–4,000 detected genes, 500–40,000 unique molecular identifiers (UMIs), and a mitochondrial transcript proportion below 20% were retained. After quality control, 36,819 cells remained, comprising 12,603 cells from the DKD group and 24,216 cells from the control group.

Data were normalized by the LogNormalize method with a scale factor of 10,000, and 3,000 highly variable genes were identified using the variance-stabilizing transformation (vst) method. Normalized data were centered and scaled with the mitochondrial transcript proportion regressed out, and principal component analysis (PCA) was performed on the highly variable genes. Fifty principal components were computed, of which the first 30 were retained for downstream analysis. Batch effects were corrected with the Harmony algorithm (Korsunsky et al., 2019). A nearest-neighbour graph was constructed from the Harmony-corrected dimensions, and unsupervised graph-based clustering was performed at a resolution of 0.5. Cell distributions were visualized by t-distributed stochastic neighbour embedding (t-SNE) and uniform manifold approximation and projection (UMAP).

Positive cluster-marker genes were identified with the FindAllMarkers function using the Wilcoxon rank-sum test, requiring expression in at least 25% of cells, an average log_2_ fold change >0.25, and an adjusted *P* value <0.05. Guided by recent single-cell transcriptomic studies of DKD (Jiang et al., 2025a; Jiang et al., 2025b), cell identities were assigned manually by comparing cluster-specific genes with canonical mouse renal and immune-cell markers; SingleR, celldex, and other automated reference-based annotation tools were not used. Representative markers included *Lrp2* and *Slc34a1* for proximal tubular cells; *Slc12a1* and *Umod* for thick ascending limb cells; *Aqp2* and *Hsd11b2* for collecting duct principal cells; *Atp6v0d2* and *Slc4a1* for collecting duct intercalated cells; *Nphs1* and *Nphs2* for podocytes; *Pecam1* and *Emcn* for endothelial cells; and *Itga8*, *Gata3*, *Pdgfrb*, and *Des* for mesangial cells. Notably, *Fn1* was explicitly excluded from all marker panels prior to annotation, ensuring that cell-type assignment was independent of the gene of interest. Canonical-marker expression was examined using dot plots, whereas *Fn1* expression was visualized across annotated cell types and experimental groups using feature and violin plots.

For the sample-level analysis of mesangial cells, raw counts were aggregated by biological sample to generate pseudobulk profiles. *Fn1* expression was normalized as log_2_ counts per million (CPM) and compared between DKD and control mice using a two-sided Student’s t-test, with each mouse treated as one biological replicate (n = 3 per group).

### 2.10 Database-based chemical–gene association mapping

The Comparative Toxicogenomics Database (CTD; https://ctdbase.org/; accessed 6 June 2026) was queried to retrieve curated chemical–gene interaction records for the candidate genes identified in the preceding analyses (Davis et al., 2025). The retrieved records were used to construct a chemical–gene association network. Information on traditional Chinese medicines (TCMs), their bioactive components, and annotated gene targets was subsequently retrieved from the Traditional Chinese Medicine Systems Pharmacology Database and Analysis Platform (TCMSP; https://tcmsp-e.com/; accessed 6 June 2026) (Ru et al., 2014). These data were integrated to construct a TCM–component–candidate gene association network. The resulting networks summarized database-reported associations and were not interpreted as evidence of direct compound–protein binding or therapeutic efficacy.

### 2.11 Surface plasmon resonance assay

Surface plasmon resonance (SPR) measurements were performed using a Biacore 1K + instrument (Cytiva, 29726018) equipped with a Series S Sensor Chip CM5 (Cytiva, BR100530). Recombinant human FN1 protein (MedChemExpress, HY-P73063) was diluted to 50 μg/mL in acetate buffer at pH 4.0 and immobilized on flow cell 3 (Fc3) through standard amine coupling. Unreacted sites were blocked with ethanolamine, yielding an immobilization level of approximately 6,053 response units (RU). The running buffer consisted of 1× PBST containing 5% DMSO. Baicalein was prepared as a twofold dilution series at concentrations ranging from 1.5625 to 100 μM, with 0 μM serving as the buffer control. Samples were injected using a multi-cycle kinetics protocol at a flow rate of 30 μL/min. Association and dissociation were monitored for 60 and 30 s, respectively. A DMSO solvent-correction procedure was applied to minimize bulk refractive-index effects. After subtraction of the 0 μM control response, sensorgrams were analysed using Biacore Insight Evaluation Software and globally fitted to a 1:1 binding model to determine the kinetic and affinity parameters.

### 2.12 External validation of candidate genes in an independent dataset

GSE47184, comprising 18 DKD and 7 normal renal tissue samples, from the GEO database was selected as the external validation dataset (Ritchie et al., 2015). The expression levels of the selected candidate genes were extracted and compared between the DKD and control groupsStatistical analysis was performed using a two-sided Wilcoxon rank-sum test.

### 2.13 Animal models and tissue sample collection

Eighteen 10-week-old specific-pathogen-free (SPF) male mice were used. Six db/m mice were assigned to the control group, whereas 12 db/db mice were randomly allocated to the untreated DKD group and the baicalein-treated DKD group (n = 6 per group). All mice were obtained from Changzhou Cavins Laboratory Animal Co., Ltd. Baicalein was obtained from Ambeed (China, catalog no A106424). Distilled water was used as the vehicle. Mice in the baicalein-treated DKD group received baicalein by oral gavage at a dose of 50 mg/kg once daily for 4 consecutive weeks. The blank control group and the model group received an equal volume of distilled water by oral gavage according to the same schedule. Two hours after the final administration, the mice were anesthetized by intraperitoneal injection of sodium pentobarbital (40 mg/kg). Blood samples were collected via the retro-orbital route, after which the mice were euthanized by cervical dislocation under anesthesia and the kidneys were rapidly harvested. A portion of each kidney was snap-frozen in liquid nitrogen for subsequent RNA and protein extraction, while the remaining tissue was fixed in 4% paraformaldehyde for histological and immunohistochemical analysis. All animal procedures were approved by the Institutional Animal Care and Use Committee of Hebei Provincial Hospital of Chinese Medicine (approval no. IACUC-HPHCM-2024072) and were conducted in a licensed laboratory animal facility in accordance with institutional guidelines for the care and use of laboratory animals.

### 2.14 Assessment of blood glucose and renal function

At the end of the 4-week intervention, blood glucose levels were measured using a portable blood glucose meter and compatible test strips. Blood samples were collected under anesthesia as described above, and serum was subsequently separated. Serum creatinine (Scr) and blood urea nitrogen (BUN) levels were measured using a biochemical analyzer. Blood glucose and BUN levels were expressed in mmol/L, whereas Scr levels were expressed in μmol/L. Six mice from each group were included as independent biological replicates.

### 2.15 Histopathological analysis

The fixed kidney tissues were dehydrated through a graded ethanol series, cleared, and embedded in paraffin. Prior to staining, the paraffin sections were deparaffinized and rehydrated, followed by haematoxylin and eosin (H&E), periodic acid–Schiff (PAS), and Masson’s trichrome staining, histopathological changes were examined and imaged under a light microscope.

### 2.16 Western blot analysis

Kidney tissues were weighed and homogenized in RIPA lysis buffer (Solarbio, R002) at a tissue-to-buffer ratio of 1:6 (w/v). The homogenates were incubated on ice for 30 min, with mixing every 10 min, and then centrifuged at 12,000 × g for 30 min at 4 °C. The supernatants were collected for subsequent analysis. Protein concentrations were determined using the Bradford assay. Protein samples were mixed with 4× loading buffer, heated for 5 min, and then cooled on ice. Equal amounts of protein were separated by 10% sodium dodecyl sulfate–polyacrylamide gel electrophoresis and transferred onto polyvinylidene difluoride (PVDF) membranes at 25 V for 30–40 min. The membranes were blocked with 5% skimmed milk for 1–2 h at room temperature and incubated overnight at 4 °C with primary antibodies against α-SMA (Affinity, AF1032), fibronectin (Affinity, AF5335), collagen I (Affinity, AF7001), and TGF-β1 (SAB, 41494), each diluted 1:700. GAPDH (BOSTER, A00227-1) was used as the loading control. After washing with TBST, the membranes were incubated with an HRP-conjugated goat anti-rabbit IgG secondary antibody (BOSTER, BA1054; 1:8,000) for 1 h at room temperature. Protein bands were visualized using an enhanced chemiluminescence reagent and captured using a Tanon 5200 chemiluminescence imaging system. Band intensities were quantified using ImageJ software and normalized to the corresponding GAPDH bands.

### 2.17 RT-qPCR analysis

Total RNA was extracted from 50 to 100 mg of kidney tissue using 1 mL of TriQuick Reagent (Solarbio, R1100) according to the manufacturer’s instructions. RNA concentration and purity were determined using a NanoVue Plus microvolume spectrophotometer (Healthcare Bio-Sciences AB, Sweden). First-strand complementary DNA (cDNA) was synthesized from 1 μg of total RNA using the SureScript First-Strand cDNA Synthesis Kit (GeneCopoeia, QP056T) in a 20-μL reaction. The reverse-transcription program consisted of 25 °C for 5 min, 42 °C for 15 min, and 85 °C for 5 min, followed by holding at 4 °C. RT-qPCR was performed using 2× SYBR Green qPCR Master Mix (No ROX; Servicebio, G3320-05) on a SLAN-96S real-time PCR system (Hongshi Medical Technology, Shanghai, China). Each 20-μL reaction contained 10 μL of qPCR master mix, 2 μL of each primer, 2 μL of cDNA, and 4 μL of nuclease-free water. The amplification program comprised an initial denaturation at 95 °C for 3 min, followed by 40 cycles at 95 °C for 15 s and 60 °C for 30 s. Primers for *Fn1*, *Acta2*, *Col1a1*, *Tgfb1*, and *Gapdh* were synthesized by Wuhan GeneCreate Biological Engineering Co., Ltd. (Wuhan, China), and their sequences are provided in Supplementary Table S1. *Gapdh* was used as the reference gene, and the relative mRNA expression levels of *Fn1*, *Acta2*, *Col1a1*, and *Tgfb1* were calculated using the 2^−ΔΔCt method.

### 2.18 Immunohistochemical analysis

Kidney tissues were fixed in 4% paraformaldehyde, processed for paraffin embedding, and sectioned at a thickness of 4 μm using a Leica RM2135 microtome (Leica, Germany). After deparaffinization and rehydration, antigen retrieval was performed under high pressure in 3% citrate antigen-retrieval solution for 10 min after the pressure valve opened. The pressure cooker was then allowed to cool naturally to room temperature. The sections were treated with 3% hydrogen peroxide in methanol for 20 min and blocked with 5% bovine serum albumin (BOSTER, AR0004) at room temperature for 30 min. The sections were incubated overnight at 4 °C with primary antibodies against α-SMA (Affinity, AF1032; 1:80), fibronectin (Affinity, AF5335; 1:80), collagen I (Affinity, AF7001; 1:70), or TGF-β1 (Abcam, ab215715; 1:60). After three 5-min washes with phosphate-buffered saline, the sections were incubated with a two-step immunohistochemical detection system (Zhongshan Golden Bridge Biotechnology, PV-6000) at 37 °C for 20 min. Following three additional 5-min washes, immunoreactivity was visualized using a DAB chromogenic kit (Zhongshan Golden Bridge Biotechnology, ZLI-9018). The sections were counterstained with hematoxylin (Zhuhai Baso Biotechnology, BA-4041). After dehydration and clearing, the sections were mounted with neutral resin (Zhongshan Golden Bridge Biotechnology, ZLI-9555). Images were acquired using an Olympus BX43 light microscope equipped with an Olympus UC90 imaging system. For quantitative analysis, DAB-positive areas were segmented using ImageJ with identical thresholding and analysis parameters across all samples. The average optical density was calculated as the integrated optical density divided by the DAB-positive area.

### 2.19 Mesangial cell culture and experimental treatments

SV40 MES 13 mouse glomerular mesangial cells were obtained from Procell (China). Cells were maintained in growth medium comprising 70.5% DMEM (Procell, PM150210), 23.5% Ham’s F-12 (Procell, PM150810), 5% fetal bovine serum (Procell, 164,210), and 1% penicillin–streptomycin (Procell, PB180120). Cells were cultured at 37 °C in a humidified atmosphere containing 5% CO_2_.

For glucose and osmotic-control experiments, normal-glucose (NG) medium contained a final D-glucose concentration of 5.5 mmol/L, whereas high-glucose (HG) medium contained 30 mmol/L D-glucose. Mannitol osmotic-control (MA) medium contained 5.5 mmol/L D-glucose and 24.5 mmol/L D-mannitol to match the osmotic conditions of the HG medium. Baicalein (Ambeed, A106424, China) was applied at a final concentration of 20 μM, as selected from the CCK-8 cell-viability assay. After transfection, cells were exposed to the designated glucose, mannitol, and baicalein conditions for 24 h before collection for subsequent RT-qPCR and Western blot analyses.

### 2.20 CCK-8 cell viability assay

Baicalein reduced SV40 MES 13 cell viability in a concentration-dependent manner after 24 h of exposure. Relative cell viability was 62.72% ± 5.31% at 20 μM and decreased to 22.14% ± 1.83% at 160 μM. Nonlinear regression of the concentration-response data yielded an estimated half-maximal inhibitory concentration (IC_50_) of 34.68 μM. Based on the concentration-response and time-course results, 20 μM baicalein and a treatment duration of 24 h were selected for subsequent experiments.

### 2.21 FN1 overexpression and rescue design

Cells were transfected with a customized mouse *Fn1*-overexpression plasmid (*Fn1*-OE; Applied Biological Materials, Canada) or the corresponding empty vector using Lipo3.0 transfection reagent (Abbkine, China; BMU111) according to the manufacturer’s instructions. At 24 h after transfection, the medium was replaced with the designated glucose, mannitol, and baicalein treatment medium, and the cells were incubated for an additional 24 h.

The rescue experiment comprised the following six groups:NG + Vector; MA + Vector; HG + Vector; HG + BAI + Vector; HG + BAI + FN1-OE; HG + FN1-OE.The comparison between HG + Vector and HG + BAI + Vector evaluated the protective effect of baicalein under high-glucose conditions. The comparison between HG + BAI + Vector and HG + BAI + FN1-OE evaluated whether FN1 overexpression attenuated the effect of baicalein. The HG + Vector versus HG + FN1-OE comparison provided supporting evidence for the effects of FN1 overexpression under high-glucose conditions. Gene and protein expression was subsequently assessed using RT-qPCR and Western blotting.

### 2.22 RT-qPCR analysis of mesangial cells

Total RNA was extracted from each experimental group using TriQuick Reagent (Solarbio, R1100) at a volume of approximately 1 mL per 10 cm^2^ of cultured cells. RNA concentration and purity were measured using a NanoVue Plus microvolume spectrophotometer. First-strand cDNA was synthesized from 1 μg of total RNA using the SureScript First-Strand cDNA Synthesis Kit (GeneCopoeia, QP056T). Each 20-μL reverse-transcription reaction was incubated at 25 °C for 5 min, 42 °C for 15 min, and 85 °C for 5 min, followed by holding at 4 °C. RT-qPCR was performed using 2× SYBR Green qPCR Master Mix (No ROX; Servicebio, G3320-05) on a SLAN-96S real-time PCR system. Each 20-μL qPCR reaction contained 10 μL of qPCR master mix, 2 μL of each primer, 2 μL of cDNA, and 4 μL of nuclease-free water. The amplification program comprised an initial denaturation at 95 °C for 3 min, followed by 40 cycles of denaturation at 95 °C for 15 s and annealing/extension at 60 °C for 30 s. Primers for *Fn1*, *Acta2*, *Col1a1*, *Tgfb1*, and *Tuba1a* were synthesized by Wuhan GeneCreate Biological Engineering Co., Ltd. (Wuhan, China), and their sequences are provided in Supplementary Table S1. *Tuba1a* was used as the internal reference gene. Relative expression levels of *Fn1*, *Acta2*, *Col1a1*, and *Tgfb1* were calculated using the 2^−ΔΔCt^ method. Three independent experiments were performed.

### 2.23 Western blot analysis of mesangial cells

Cells were washed once with phosphate-buffered saline and lysed with RIPA buffer (Solarbio, R002) at 1 mL per well. Cells were lysed at 4 °C for 30 min and collected by repeated pipetting. Protein concentrations were measured using a Bradford assay kit (BOSTER, AR0145). Protein samples were mixed with 4× loading buffer, heated for 5 min, and cooled on ice for 5 min. Equal amounts of total protein were separated by 10% SDS-PAGE. Proteins were transferred onto PVDF membranes (Millipore, IPVH00010) at 25 V for 30–40 min. The membranes were blocked with 5% skimmed milk at room temperature for 1–2 h. They were then incubated overnight at 4 °C with primary antibodies diluted 1:800. The antibodies recognized α-SMA (Proteintech, 14395-1-AP), fibronectin (Proteintech, 15613-1-AP), collagen I (SAB, #46888), TGF-β1 (Proteintech, 81746-2-RR), or α-tubulin (Proteintech, 66031-1-Ig). After washing, the membranes were incubated for 1 h at room temperature with the appropriate HRP-conjugated secondary antibody. HRP-conjugated anti-rabbit IgG (BOSTER, BA1054) or HRP-conjugated anti-mouse IgG was used at 1:8,000. Protein bands were visualized using an enhanced chemiluminescence reagent (Abbkine, BMU102-CN) and a chemiluminescence imaging system. Band intensities were quantified using ImageJ and normalized to the corresponding α-tubulin bands. The normalized value of the NG + Vector group was set to 1. Three independent experiments were performed.

### 2.24 Statistical analysis

All statistical analyses and graphical representations were performed using GraphPad Prism 10.4.1. Experimental results are expressed as Mean ± SD. Differences between groups were assessed using one-way or two-way ANOVA; for *post hoc* multiple comparisons, the Tukey method was used for data with homogeneous variances, and the Games-Howell method was used for data with heterogeneous variances. Differences were considered statistically significant when P < 0.05.

## 3 Results

### 3.1 Integrated transcriptomic analysis and identification of the DKD-Associated co-expression module

**FIGURE 2:**
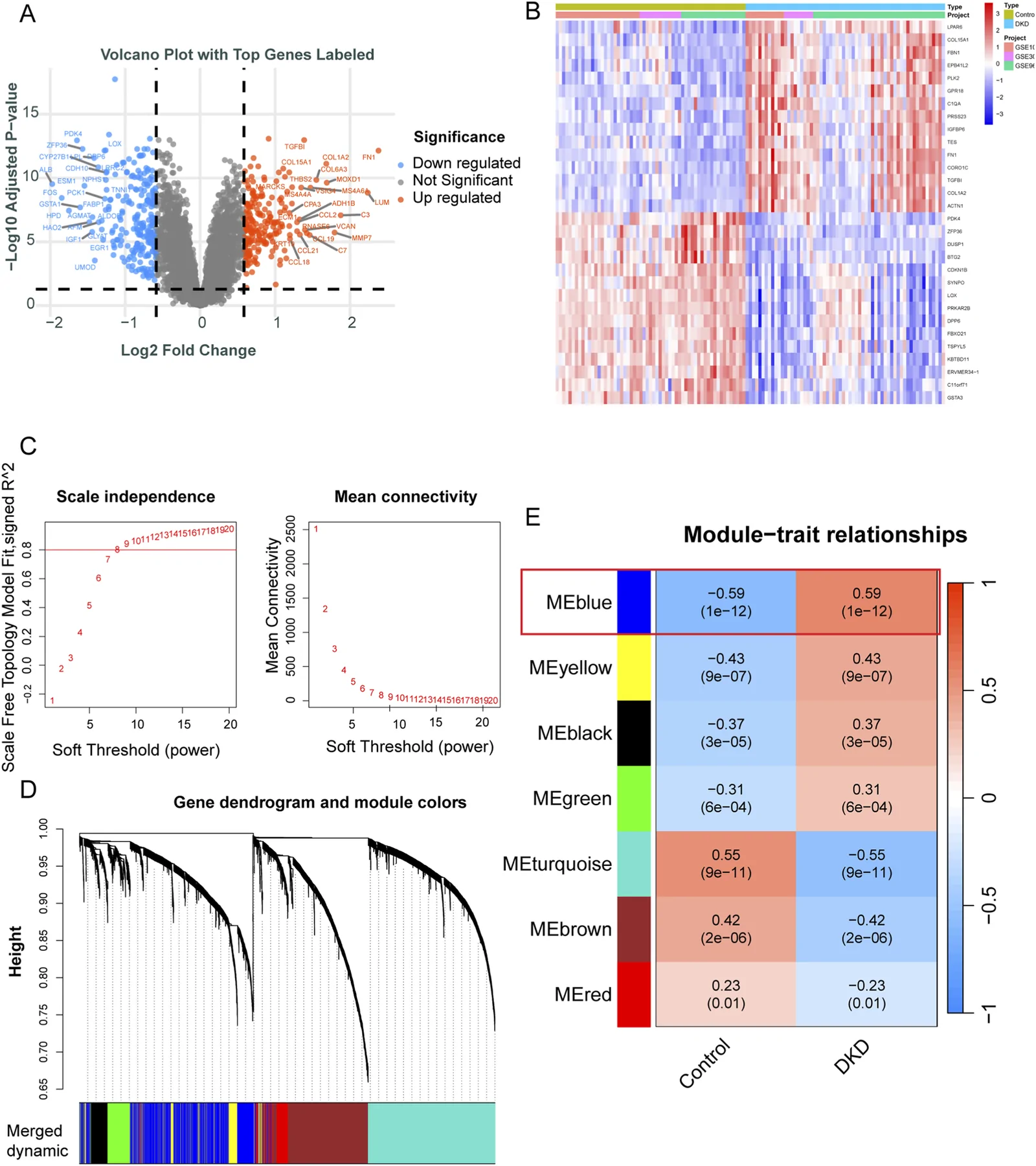
Differential expression analysis and identification of the DKD-associated WGCNA module. **(A)** Volcano plot showing differentially expressed genes (DEGs) between 62 DKD and 59 control renal tissue samples. Dashed lines indicate the thresholds of |log_2_ fold change| > 0.585 and false discovery rate-adjusted *P* < 0.05. Representative genes are labelled. **(B)** Heatmap showing the 30 most significant DEGs, comprising 15 upregulated and 15 downregulated genes ranked by adjusted *P* value. Sample annotations indicate the disease status and source dataset. **(C)** Scale-free topology fit and mean connectivity analyses supporting the selection of a soft-thresholding power of 8 for weighted gene co-expression network analysis (WGCNA). **(D)** Hierarchical clustering dendrogram of genes with the corresponding merged module assignments. **(E)** Heatmap showing correlations between module eigengenes and the control or DKD traits. The red outline highlights the blue module, which contained 1,346 genes and showed the strongest positive correlation with DKD (*r* = 0.59, *P* = 1 × 10^−12^). Together, these results defined DKD-associated transcriptional alterations and identified the blue module as a disease-related co-expression module for downstream screening.

The GSE30528, GSE96804, and GSE104948 datasets were normalized, corrected for batch effects, and integrated into a training cohort comprising 62 DKD and 59 control renal tissue samples. Boxplots and principal component analysis showed reduced inter-dataset variation after batch-effect correction (Supplementary Figure S1). Using |log_2_ fold change| > 0.585 and false discovery rate-adjusted *P* < 0.05, we identified 441 differentially expressed genes (DEGs). Among them, 197 genes were upregulated and 244 were downregulated in DKD (Figure 2A). A heatmap of the 30 most significant DEGs, comprising 15 upregulated and 15 downregulated genes, showed distinct expression patterns between DKD and control samples (Figure 2B). Scale-free topology fit and mean connectivity analyses supported the selection of a soft-thresholding power of 8 for weighted gene co-expression network analysis (Figure 2C). Hierarchical clustering separated the genes into distinct co-expression modules (Figure 2D). The blue module contained 1,346 genes and showed the strongest positive correlation with DKD (*r* = 0.59, *P* = 1 × 10^−12^; Figure 2E). Therefore, the blue module was selected for subsequent intersection analysis.

### 3.2 Identification of intersecting candidate genes and ECM-related functional enrichment

**FIGURE 3:**
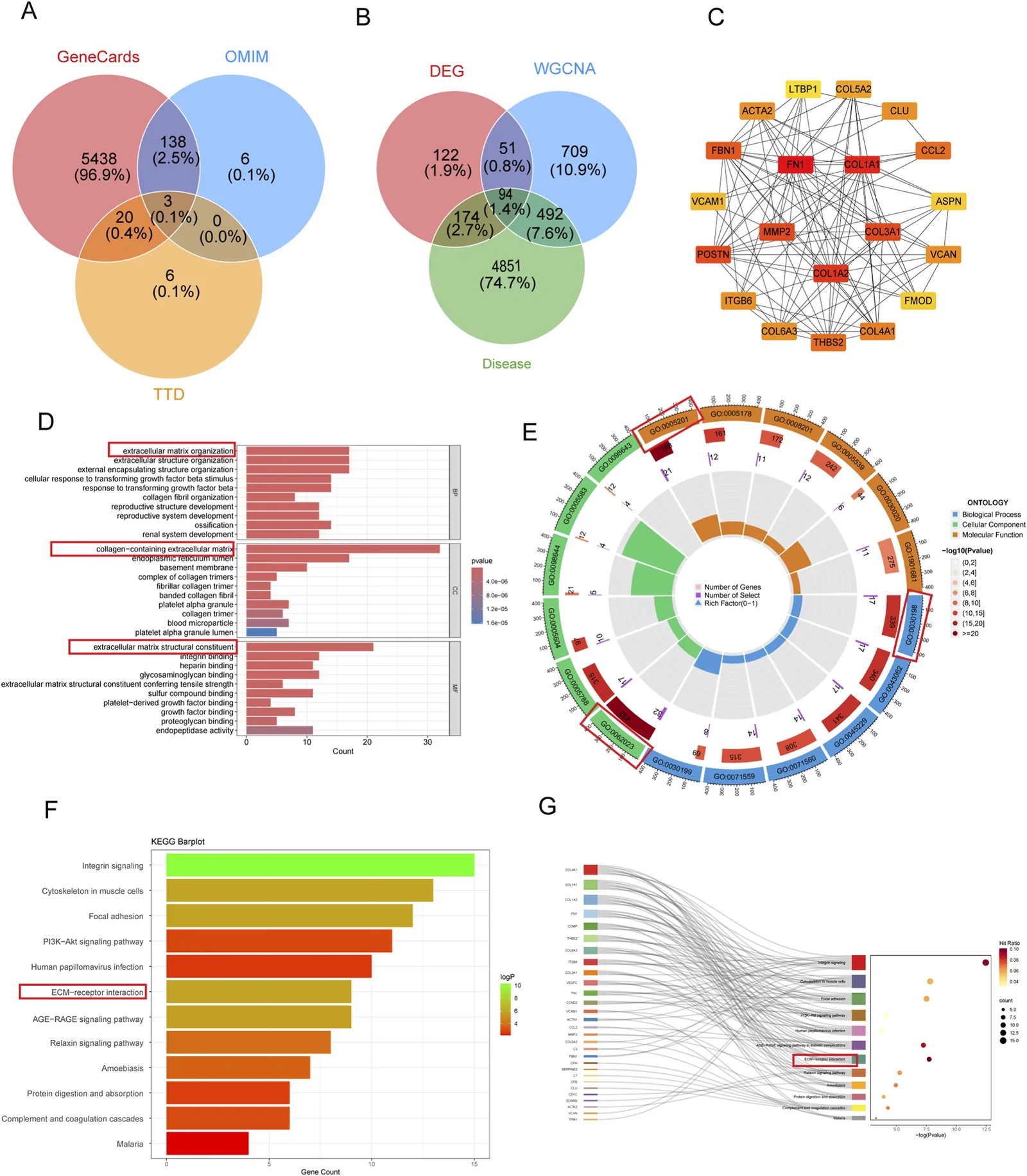
Identification of intersecting candidate genes and ECM-related functional enrichment. **(A)** Venn diagram showing DKD-associated genes retrieved from GeneCards, OMIM, and TTD. The union of the three databases yielded 5,611 nonredundant genes. **(B)** Venn diagram showing the intersection of 441 DEGs, 1,346 genes from the blue WGCNA module, and 5,611 DKD-associated genes. Ninety-four genes were shared among the three sets. **(C)** Degree-based protein–protein interaction network constructed in STRING using *Homo sapiens* and a high-confidence score of 0.700. Disconnected nodes were hidden, and the 20 most highly connected genes were retained. Node colours range from yellow to red with increasing degree. **(D)** Bar plots showing the leading Gene Ontology terms in the biological-process, cellular-component, and molecular-function categories. Bar length represents the number of enriched genes, and colour indicates the enrichment *P* value. Red boxes highlight extracellular matrix organization, collagen-containing extracellular matrix, and extracellular matrix structural constituent. **(E)** Circular Gene Ontology enrichment plot summarizing enriched terms, gene counts, selected gene numbers, rich factors, and statistical significance. Red boxes highlight GO:0030198, GO:0062023, and GO:0005201. **(F)** KEGG bar plot showing the leading enriched pathways. Bar length represents gene count, and colour represents statistical significance. The red box highlights ECM–receptor interaction. **(G)** Sankey–bubble plot showing relationships between candidate genes and enriched KEGG pathways. Bubble size represents gene count, while bubble colour indicates the hit ratio. The red box highlights ECM–receptor interaction. The PPI network further revealed the interactions among the intersecting candidate genes, and the 20 most highly connected genes were retained for subsequent machine-learning analysis.

Integration of the GeneCards, OMIM, and TTD results yielded 5,611 nonredundant DKD-associated genes (Figure 3A). Their intersection with the 441 DEGs and 1,346 genes in the blue WGCNA module identified 94 shared candidate genes (Figure 3B). A protein–protein interaction network was constructed in STRING using *Homo sapiens* and a high-confidence score of 0.700. Disconnected nodes were hidden, and degree-based ranking retained the 20 most highly connected genes for visualization (Figure 3C). *FN1* showed the highest connectivity and was accompanied by multiple extracellular matrix-related genes, including *COL1A1*, *COL1A2*, *COL3A1*, *COL4A1*, *FBN1*, and *VCAN*. Gene Ontology enrichment consistently indicated extracellular matrix involvement (Figures 3D,E). The leading terms included extracellular matrix organization in the biological-process category (GO:0030198; FDR-adjusted *P* = 7.78 × 10^−9^), collagen-containing extracellular matrix in the cellular-component category (GO:0062023; FDR-adjusted *P* = 2.32 × 10^−26^), and extracellular matrix structural constituent in the molecular-function category (GO:0005201; FDR-adjusted *P* = 4.80 × 10^−20^). KEGG enrichment further identified integrin signaling, ECM–receptor interaction, and focal adhesion among the prominent pathways (Figures 3F,G). Their FDR-adjusted *P* values were 5.26 × 10^−11^, 9.53 × 10^−7^, and 1.19 × 10^−6^, respectively. The ECM–receptor interaction pathway contained nine candidate genes, including *FN1*, *COL1A1*, *COL1A2*, *COL4A1*, *COL6A3*, *THBS2*, *TNC*, *COMP*, and *ITGB6*.

### 3.3 Machine-learning screening and identification of candidate diagnostic markers

**FIGURE 4:**
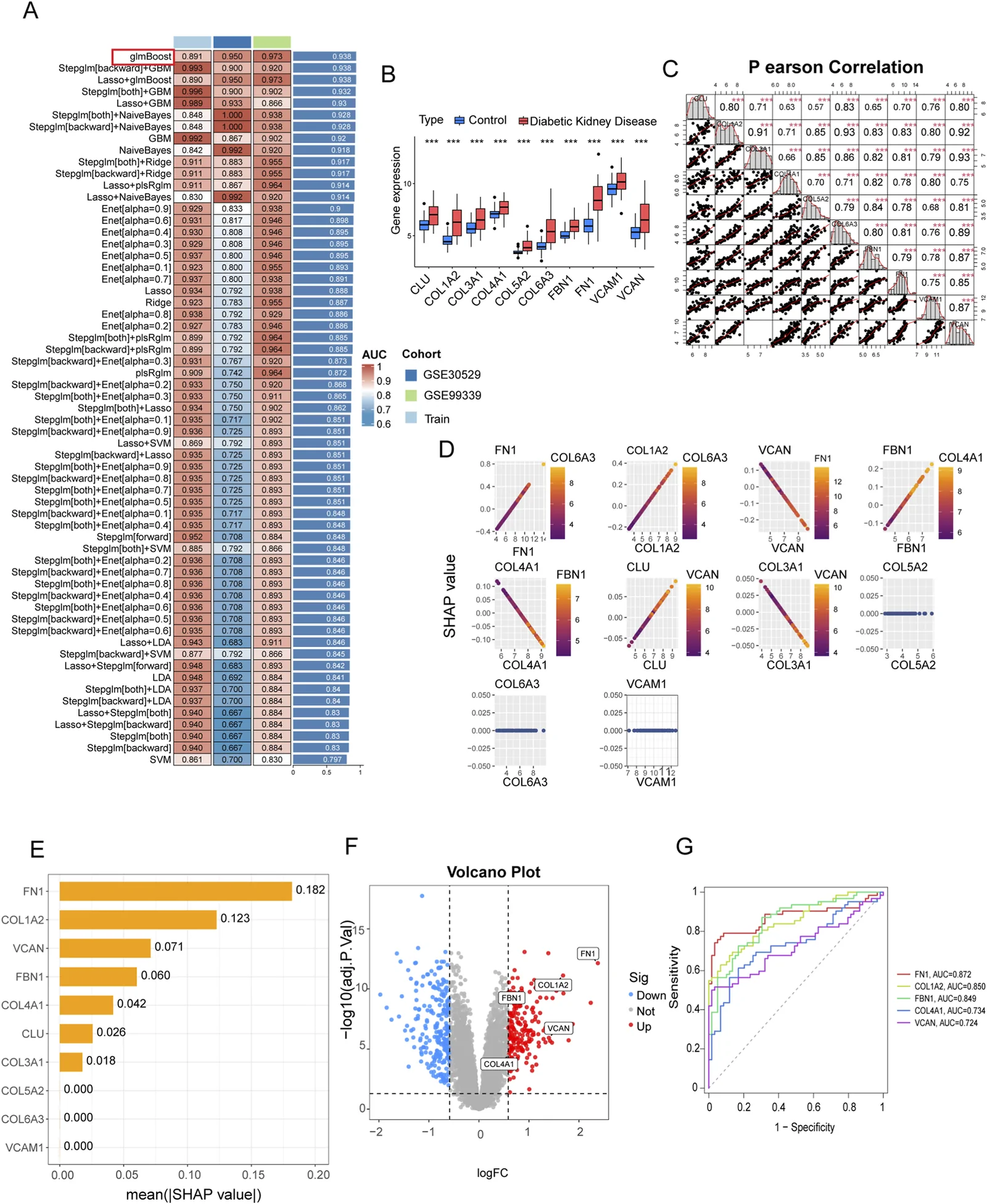
Machine-learning screening and SHAP-based identification of candidate diagnostic markers for DKD. **(A)** Heatmap showing the AUCs of 61 retained machine-learning models in the training set and the GSE30529 and GSE99339 internal validation cohorts. Models containing five or fewer features were excluded. The red box indicates the glmBoost model, which achieved AUCs of 0.891, 0.950, and 0.973, respectively, and the highest mean AUC of 0.938. **(B)** Expression distributions of the 10 genes retained by the glmBoost model in control and DKD samples. Group differences were assessed using the Wilcoxon rank-sum test; *** indicates *P* < 0.001. **(C)** Pearson correlation matrix of the 10 model features in DKD samples. Numbers indicate Pearson correlation coefficients. **(D)** SHAP dependence plots showing the feature-wise relationships between gene expression and SHAP values. **(E)** Mean absolute SHAP values of the 10 model features. The five highest-ranking genes were *FN1*, *COL1A2*, *VCAN*, *FBN1*, and *COL4A1*. **(F)** Volcano plot showing the differential expression of the five SHAP-selected genes. Red and blue points indicate upregulated and downregulated genes, respectively. **(G)** ROC curves of the five candidate diagnostic markers in the combined expression dataset. Together, these analyses identified *FN1*, *COL1A2*, *VCAN*, *FBN1*, and *COL4A1* as candidate diagnostic markers for subsequent analysis.

The 20 genes with the highest connectivity in the PPI network were used as the initial candidate pool for machine-learning analysis. A total of 113 algorithmic combinations were constructed, of which 61 models containing more than five features were retained for performance evaluation. Among these models, glmBoost achieved the highest mean AUC across the training set and the two internal validation cohorts, with AUCs of 0.891, 0.950, and 0.973 in the training set, GSE30529, and GSE99339, respectively, yielding a mean AUC of 0.938 (Figure 4A). The optimal glmBoost model retained 10 genes, namely, *CLU*, *COL1A2*, *COL3A1*, *COL4A1*, *COL5A2*, *COL6A3*, *FBN1*, *FN1*, *VCAM1*, and *VCAN*. All 10 genes were expressed at significantly higher levels in DKD samples than in control samples (all *P* < 0.001; Figure 4B), and Pearson correlation analysis revealed predominantly positive correlations among these features in DKD samples (Figure 4C). SHAP analysis was subsequently performed to quantify the contribution of each feature to the glmBoost model (Figures 4D,E). The five leading features were *FN1* (mean |SHAP value| = 0.182), *COL1A2* (0.123), *VCAN* (0.071), *FBN1* (0.060), and *COL4A1* (0.042). These genes were also significantly upregulated in the differential expression analysis (Figure 4F). ROC analysis of the combined expression dataset yielded AUCs of 0.872 for *FN1*, 0.850 for *COL1A2*, 0.849 for *FBN1*, 0.734 for *COL4A1*, and 0.724 for *VCAN* (Figure 4G). Thus, machine-learning and SHAP analyses identified these five genes as candidate diagnostic markers for subsequent analysis.

### 3.4 Reverse network pharmacology and external validation of *FN1*

**FIGURE 5:**
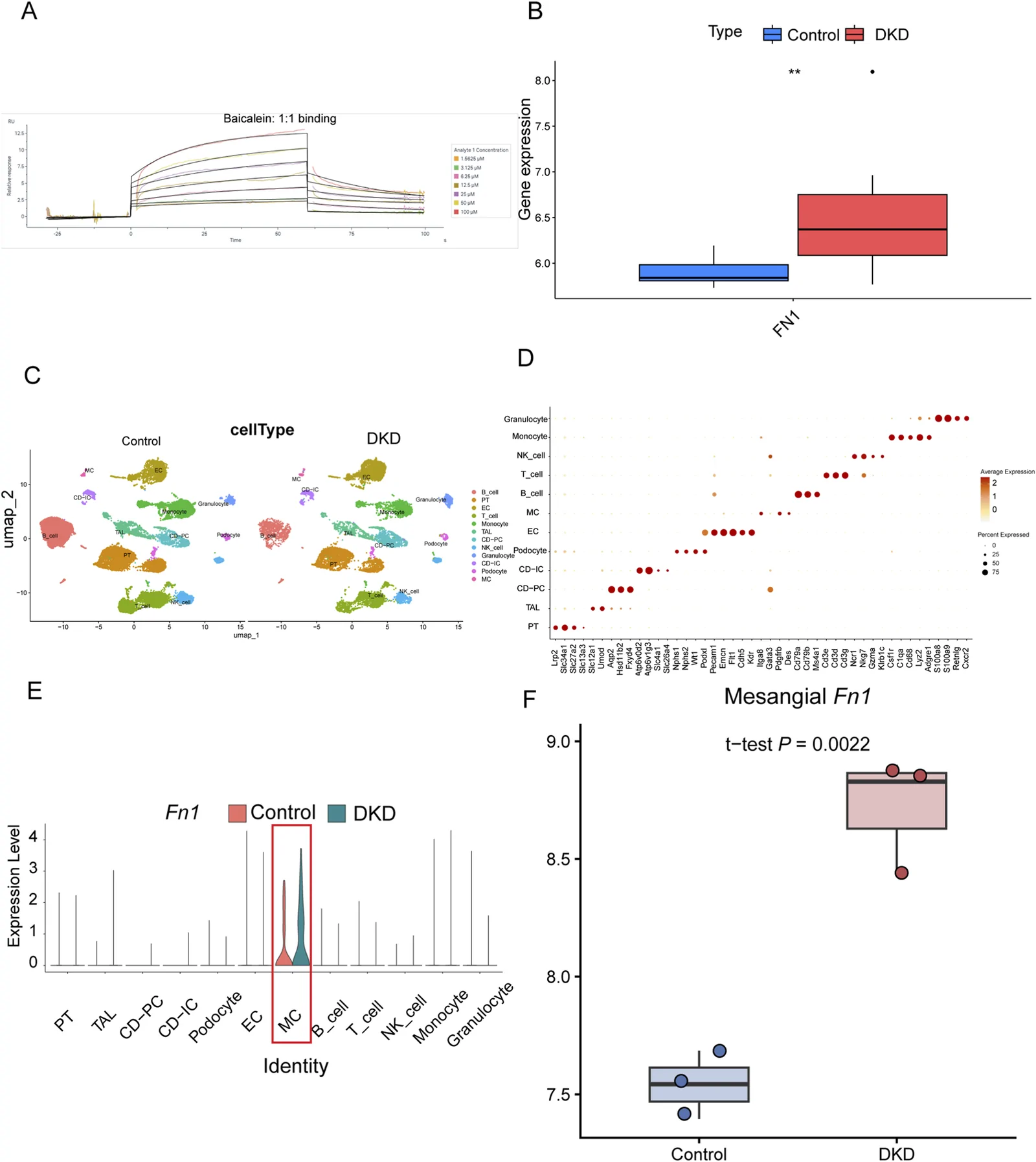
SPR analysis of the baicalein–FN1 interaction, external validation, and single-cell localization of *FN1/Fn1*. **(A)** SPR sensorgrams showing the concentration-dependent interaction between baicalein and immobilized recombinant human FN1. Coloured traces represent the experimental responses to baicalein concentrations ranging from 1.5625 to 100 μM, whereas black traces represent global fits to a 1:1 binding model. The apparent KD was 8.04 × 10^−5^ M (80.4 μM). **(B)** External validation of *FN1* expression in GSE47184, comprising 7 control and 18 DKD samples. The Wilcoxon rank-sum test was used; ** indicates *P* < 0.01. **(C)** UMAP plots showing the annotated kidney cell populations in control and DKD samples from GSE335999. **(D)** Dot plot of canonical marker genes used for manual cell-type annotation. Dot size represents the percentage of cells expressing each marker, and colour represents average expression. **(E)** Violin plots showing the descriptive distribution of *Fn1* expression across cell types. The mesangial-cell population is highlighted. **(F)** Sample-level pseudobulk comparison of mesangial-cell *Fn1* expression between control and DKD mice. Each point represents one mouse (n = 3 mice per group), and expression is presented as log2 CPM. The two-sample t-test yielded *P* = 0.0022. Together, these analyses confirmed increased *FN1/Fn1* expression in an independent bulk-transcriptomic cohort and localized the increase to mesangial cells in the mouse kidney.

Using a reverse network pharmacology strategy, we first queried the CTD database for human chemical–gene interactions involving five SHAP-selected candidate diagnostic markers: FN1, COL1A2, VCAN, FBN1, and COL4A1. The retrieved compounds were subsequently mapped to the TCMSP database to identify traditional Chinese medicine (TCM) ingredients meeting the pharmacokinetic criteria of oral bioavailability (OB) ≥30% and drug-likeness (DL) ≥0.18. No eligible TCM ingredients were identified for COL1A2, VCAN, FBN1, or COL4A1, whereas baicalein was retained as a candidate natural compound associated with FN1. Baicalein was mapped to 12 TCM sources, including Huaijiao, Cheqiancao, Dengzhanxixin, Huangjing, Banxia, Muhudie, Niuxi, Honghua, Banzhilian, Chishao, Guanhuangbo, and Huangqin. These database-derived relationships did not themselves demonstrate direct physical binding or functional target engagement between baicalein and FN1. To examine this association experimentally, we performed SPR analysis. Baicalein showed a concentration-dependent interaction with immobilized recombinant human FN1, with an apparent *K*D of 8.04 × 10^−5^ M (80.4 μM), indicating a direct micromolar-affinity interaction (Figure 5A).

The expression of *FN1* was subsequently evaluated in the independent external cohort GSE47184, which comprised 18 DKD samples and 7 controls. *FN1* expression was significantly higher in the DKD group than in the control group (*P* < 0.01; Figure 5B), independently confirming the direction of differential expression observed in the discovery cohorts.

### 3.5 Single-cell analysis localizes increased *Fn1* expression to mesangial cells

Single-cell RNA sequencing data from GSE335999 included kidney samples from three control db/m mice and three DKD db/db mice. After quality control, 36,819 cells were retained, comprising 24,216 control cells and 12,603 DKD cells. Unsupervised clustering and manual annotation using canonical markers identified 12 major renal and immune cell types (Figures 5C,D). These comprised proximal tubular cells, thick ascending limb cells, collecting duct principal cells, collecting duct intercalated cells, endothelial cells, podocytes, mesangial cells, B cells, T cells, NK cells, monocytes, and granulocytes. Additional quality-control metrics, sample-level PCA, canonical-marker expression, t-SNE visualization, and group-level cell-type proportions are presented in Supplementary Figure S3. Violin plots descriptively showed prominent *Fn1* expression in mesangial cells (Figure 5E). To avoid treating individual cells as independent biological replicates, group differences were formally evaluated using sample-level pseudobulk expression in mesangial cells. Each point represented 1 mouse, with three biological replicates per group. Mesangial-cell *Fn1* expression was significantly higher in DKD mice than in control mice (P = 0.0022; Figure 5F). These results localized the DKD-associated increase in *Fn1* expression to the mesangial-cell compartment.

### 3.6 Baicalein improves metabolic and renal functional indices and attenuates renal histopathological injury in DKD mice

**FIGURE 6:**
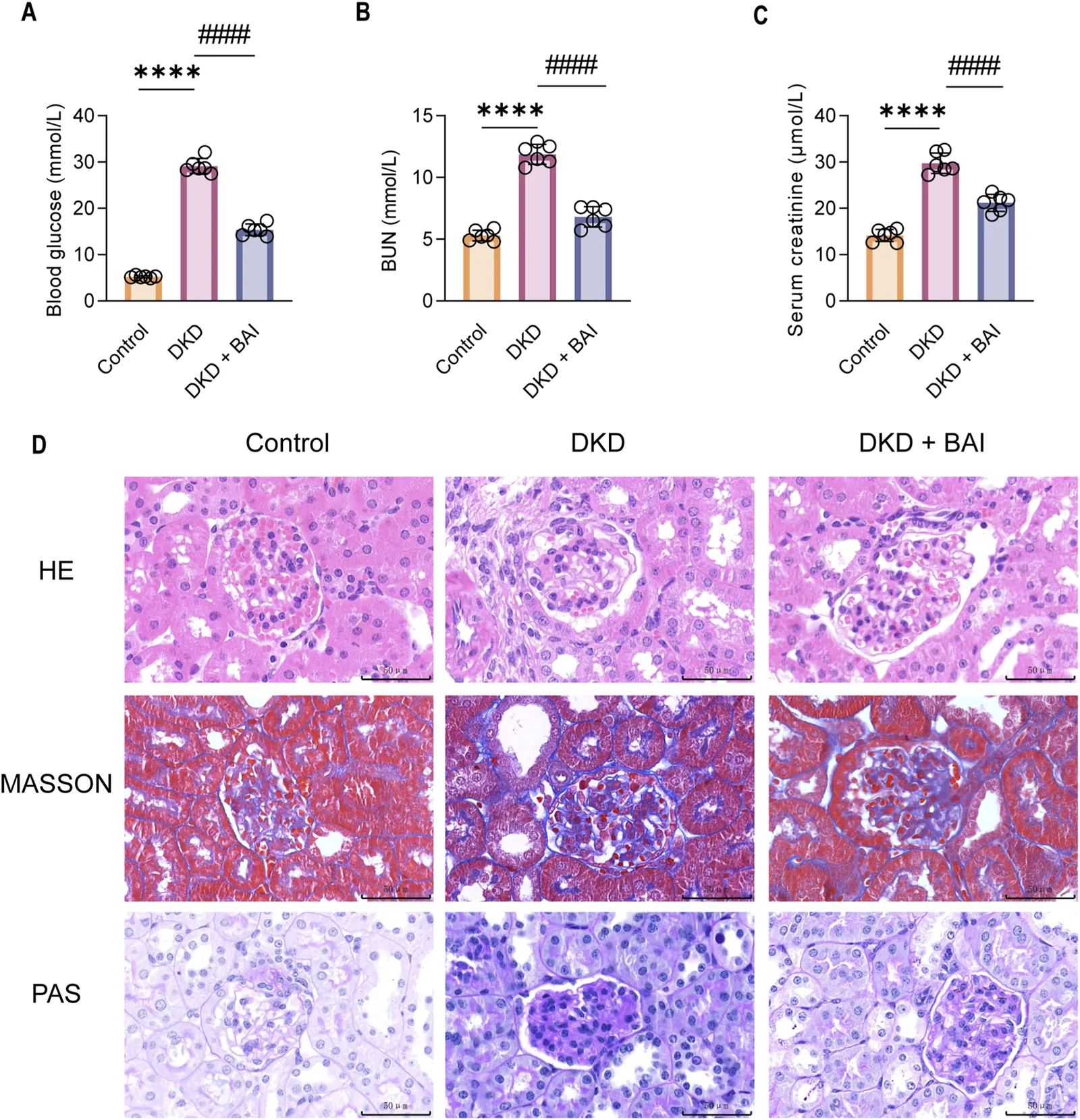
Baicalein improves metabolic and renal functional indices and attenuates renal histopathological injury in DKD mice. **(A–C)** Blood glucose, blood urea nitrogen (BUN), and serum creatinine (Scr) levels in the Control, DKD, and DKD + BAI groups. Data are presented as the mean ± SD, with each point representing one mouse (n = 6 mice per group). Statistical significance was assessed using one-way ANOVA with multiple-comparison testing. **** indicates *P* < 0.0001 versus the Control group; #### indicates *P* < 0.0001 versus the DKD group. (D) Representative H&E, Masson’s trichrome, and PAS-stained kidney sections. Scale bars, 50 μm. BAI, baicalein.

To evaluate the effects of baicalein *in vivo*, db/db mice were treated with baicalein for 4 weeks. Compared with control mice, untreated DKD mice exhibited marked increases in blood glucose, BUN, and Scr levels (all *P* < 0.0001). Baicalein treatment significantly reduced all three indices compared with untreated DKD mice (all *P* < 0.0001; Figures 6A–C). Histopathological examination further showed glomerular structural abnormalities, mesangial matrix expansion, and increased collagen deposition in the kidneys of DKD mice. These pathological changes were visibly attenuated following baicalein treatment, as shown by H&E, Masson’s trichrome, and PAS staining (Figure 6D). Collectively, these findings showed that baicalein lowered blood glucose, improved renal functional indices, and reduced renal structural injury in db/db mice.

### 3.7 Baicalein suppresses renal extracellular matrix accumulation and fibrosis-associated molecular changes

**FIGURE 7:**
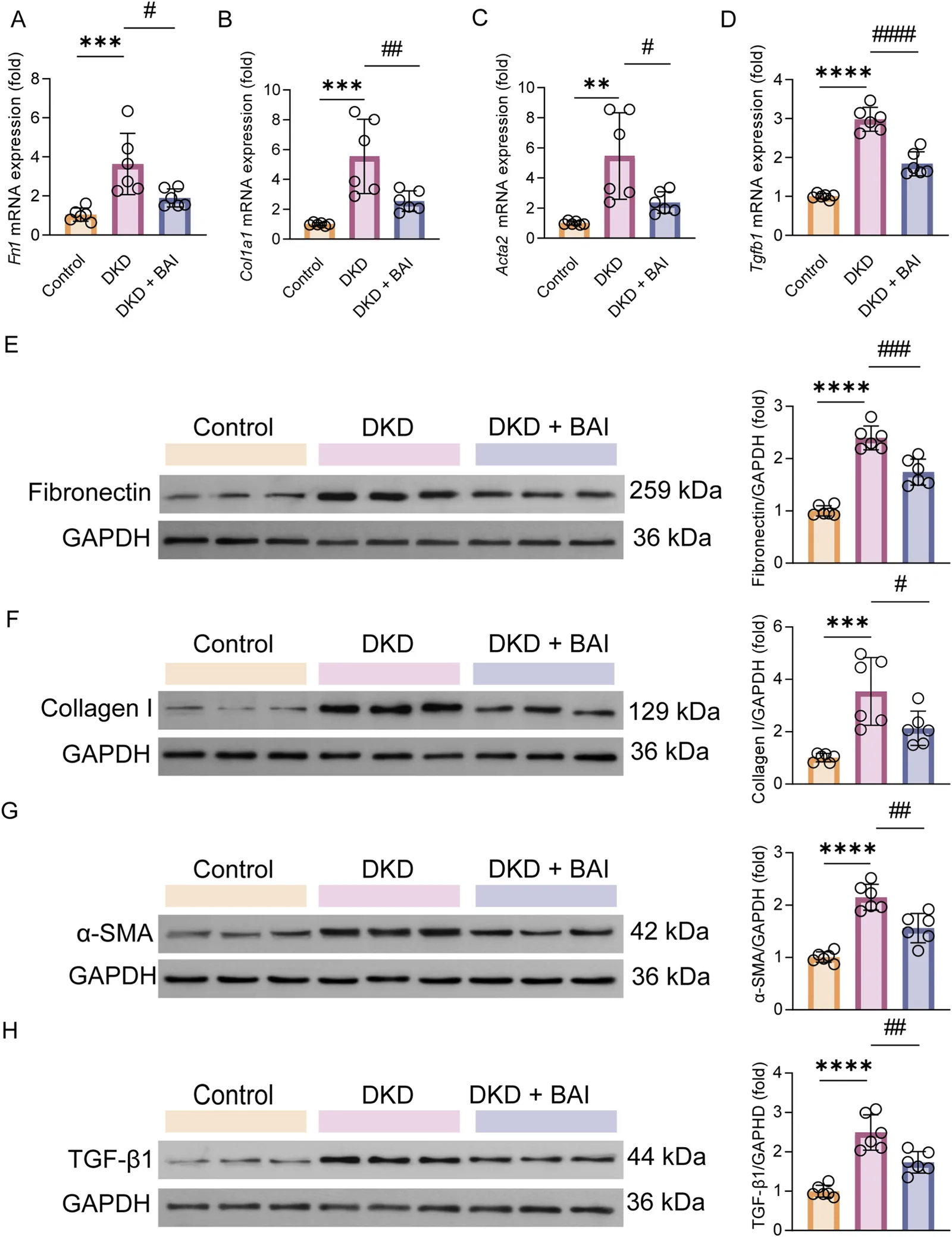
Baicalein reduces the renal expression of extracellular matrix and fibrosis-associated genes and proteins in DKD mice. **(A–D)** RT-qPCR analysis of renal *Fn1*, *Acta2*, *Col1a1*, and *Tgfb1* mRNA expression. Expression was normalized to *Gapdh* and presented relative to the Control group. **(E–H)** Representative western blots and densitometric quantification of fibronectin, collagen I, α-SMA, and TGF-β1. Protein abundance was normalized to GAPDH and presented relative to the Control group. Fibronectin, collagen I, α-SMA, TGF-β1, and GAPDH were detected at approximately 259, 129, 42, 44, and 36 kDa, respectively. Data are presented as the mean ± SD, with each point representing one independent mouse (n = 6 mice per group). Statistical significance was assessed using one-way ANOVA followed by Tukey’s multiple-comparisons test. Asterisks indicate comparisons with the Control group: *P* < 0.05, P < 0.01, *P* < 0.001, and P < 0.0001. Hash symbols indicate comparisons with the DKD group: #*P* < 0.05, ##*P* < 0.01, ###*P* < 0.001, and ####*P* < 0.0001. Uncropped Western blot images for all six mice are provided in Supplementary Figures S4, S5.

**FIGURE 8:**
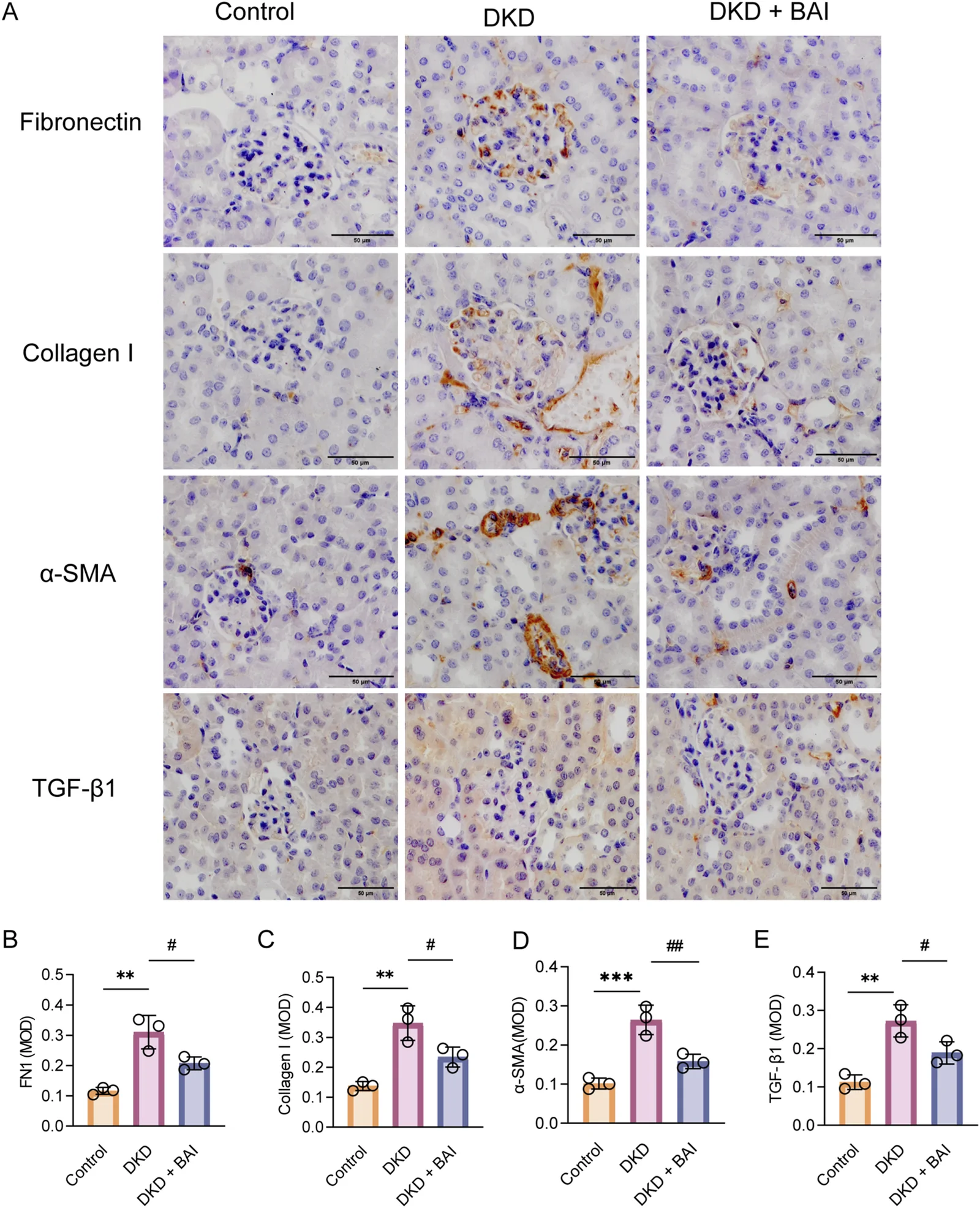
Immunohistochemical assessment of extracellular matrix and fibrosis-associated proteins in DKD mouse kidneys. **(A)** Representative immunohistochemical images of fibronectin, collagen I, α-SMA, and TGF-β1 in kidney sections from the Control, DKD, and DKD + BAI groups. Brown staining indicates DAB-positive protein expression, and nuclei were counterstained blue with haematoxylin. Scale bars, 50 μm. **(B–E)** Average optical density (AOD) of fibronectin, collagen I, α-SMA, and TGF-β1 staining, calculated as integrated optical density divided by the DAB-positive area. The same threshold and analysis parameters were applied to all images. Data are presented as the mean ± SD, with each point representing one independent mouse (n = 3 mice randomly selected from each group of six). Statistical significance was assessed using one-way ANOVA followed by Tukey’s multiple-comparisons test. Asterisks indicate comparisons with the Control group, and hash symbols indicate comparisons with the DKD group: #*P* < 0.05, ##*P* < 0.01, **P < 0.01, and ***P < 0.001. BAI, baicalein. Together, these results showed that baicalein reduced renal accumulation of fibronectin, collagen I, α-SMA, and TGF-β1 in DKD mice.

RT-qPCR analysis showed that the renal expression of *Fn1*, *Acta2*, *Col1a1*, and *Tgfb1* was significantly increased in DKD mice compared with control mice. Baicalein treatment significantly reduced the expression of all four genes relative to untreated DKD mice (Figures 7A–D). Consistent with the transcriptional results, Western blotting showed increased renal levels of fibronectin, collagen I, α-SMA, and TGF-β1 in DKD mice, whereas baicalein treatment significantly reduced the abundance of all four proteins (Figures 7E–H). Immunohistochemical staining provided spatial evidence for these molecular changes. DKD kidneys exhibited increased DAB-positive staining for fibronectin, collagen I, α-SMA, and TGF-β1. Quantification of the average optical density confirmed that the staining of all four proteins was significantly increased in DKD mice and significantly reduced following baicalein treatment (Figures 8A–E). Together, the RT-qPCR, Western blotting, and immunohistochemical results consistently showed that baicalein attenuated renal extracellular matrix accumulation and fibrosis-associated molecular changes in DKD mice.

### 3.8 *FN1* overexpression partially attenuates the effects of baicalein in high-glucose-treated mesangial cells

CCK-8 assays were first performed to determine an appropriate concentration of baicalein for subsequent cell experiments. Based on the concentration- and time-response profiles and an estimated half-maximal inhibitory concentration (IC_50_) of 34.68 μM, 20 μM baicalein was selected for subsequent experiments (Supplementary Figure S2).

**FIGURE 9:**
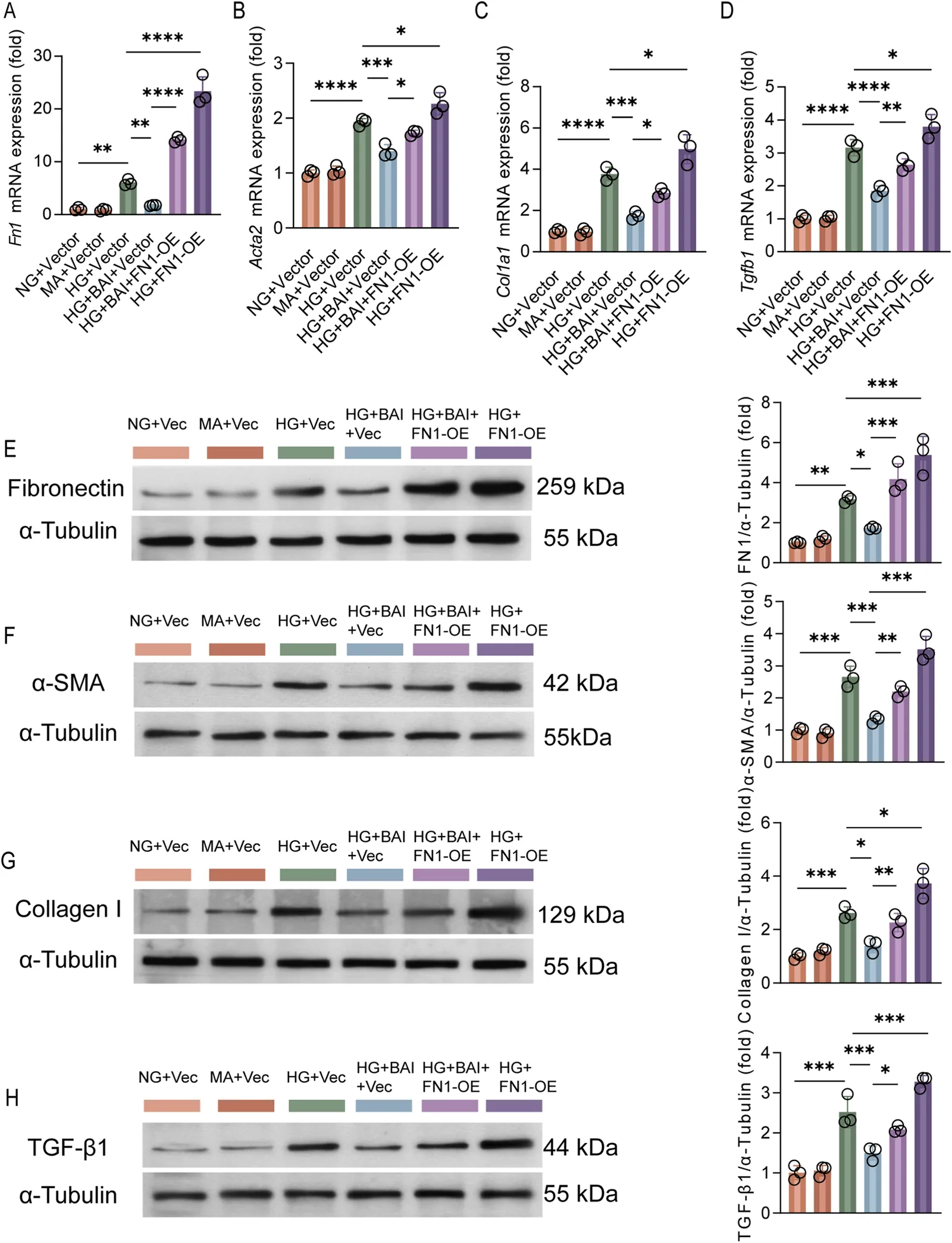
FN1 overexpression promotes a profibrotic phenotype and partially attenuates the effects of baicalein on extracellular matrix remodeling in high-glucose-treated mesangial cells. **(A–D)** RT-qPCR analysis of Fn1, Acta2, Col1a1, and Tgfb1 expression in the NG + Vector, MA + Vector, HG + Vector, HG + BAI + Vector, HG + BAI + FN1-OE, and HG + FN1-OE groups. Expression was normalized to *Tuba1a* and is presented relative to the NG + Vector group. **(E–H)** Representative western blots and densitometric quantification of fibronectin, α-SMA, collagen I, and TGF-β1. Protein abundance was normalized to α-tubulin and is presented relative to the NG + Vector group. Fibronectin, α-SMA, collagen I, TGF-β1, and α-tubulin were detected at approximately 259, 42, 129, 44, and 55 kDa, respectively. Data are presented as the mean ± SD from three independent experiments. Comparisons are indicated by connecting lines; *P < 0.05, **P < 0.01, ***P < 0.001, and ****P < 0.0001. NG, normal glucose; MA, mannitol osmotic control; HG, high glucose; BAI, baicalein; FN1-OE, FN1 overexpression; Vector, empty vector. Uncropped Western blot images with molecular-weight markers and lane annotations are provided in Supplementary Figure S5.

To examine whether FN1 contributes to the effects of baicalein on mesangial-cell extracellular matrix remodeling, a six-group FN1-overexpression rescue experiment was performed. The NG + Vector and MA + Vector groups showed comparable expression patterns, indicating that the high-glucose responses were not attributable solely to osmotic changes. Compared with NG + Vector, HG + Vector markedly increased the mRNA expression of Fn1, Acta2, Col1a1, and Tgfb1. Baicalein treatment significantly reduced the high-glucose-induced expression of all four genes. However, FN1 overexpression in baicalein-treated cells significantly increased their expression relative to HG + BAI + Vector, thereby partially attenuating the effects of baicalein (Figures 9A–D).

Western blotting showed a corresponding pattern at the protein level. High glucose increased the abundance of fibronectin, α-SMA, collagen I, and TGF-β1, whereas baicalein reduced the levels of these proteins. FN1 overexpression significantly restored fibronectin expression and partially restored α-SMA, collagen I, and TGF-β1 levels in baicalein-treated cells (Figures 9E–H). These findings further indicated that FN1 overexpression promoted a profibrotic phenotype in high-glucose-exposed mesangial cells. Overall, the rescue experiments support the involvement of FN1 in the effects of baicalein on mesangial-cell extracellular matrix remodeling. However, these results do not establish FN1 as the sole mediator or a direct binding target of baicalein.

## 4 Discussion

The principal finding of this study is that baicalein attenuated renal injury, extracellular matrix (ECM) accumulation, and the profibrotic molecular phenotype in experimental diabetic kidney disease (DKD). Cross-cohort transcriptomic integration identified a disease-associated gene set enriched in ECM-related processes. Machine-learning comparison selected glmBoost as the final model. Subsequent SHAP analysis ranked *FN1* among its most informative features, while the model retained favorable discrimination in two external validation datasets. Independently generated mouse single-cell RNA-sequencing data further provided cell-resolved validation. Sample-level pseudobulk analysis, using each mouse as the statistical unit, demonstrated increased mesangial *Fn1* expression in DKD. *In vivo* and cellular experiments subsequently showed that baicalein reduced Fibronectin and fibrosis-associated molecules. Moreover, *FN1* overexpression partially attenuated these cellular effects. SPR further demonstrated a concentration-dependent interaction between baicalein and recombinant human FN1, with an apparent *K*D of 8.04 × 10^−5^ M Collectively, these findings identify FN1/Fibronectin as one functionally supported contributing axis within the broader ECM response to baicalein. The SPR results support a direct *in vitro* interaction under the tested conditions but do not establish cellular or *in vivo* target engagement or identify FN1 as the exclusive mediator of baicalein activity.

Recent transcriptomic and multi-omics studies have repeatedly identified *FN1* among genes associated with DKD, renal fibrosis, or declining renal function (Dou et al., 2023; Lin et al., 2025; Yang Y. et al., 2025). These studies support the reproducibility of the association between *FN1* and DKD across different datasets and analytical settings. Recent DKD investigations have also combined machine learning with single-cell or spatially resolved transcriptomic analyses to improve the cellular interpretation of computationally selected genes (Jiang et al., 2025a; Jiang et al., 2025b). However, computational prioritization alone cannot determine whether altered *FN1* expression is merely a marker of matrix accumulation or an active contributor to the profibrotic phenotype. The present study extends this evidence by integrating cross-cohort external validation with independently generated mouse single-cell transcriptomic data, *in vivo* pharmacological evidence, and *FN1* overexpression experiments. FN1 was therefore selected not as an exclusive mediator of baicalein activity, but as a convergently prioritized and experimentally supported component of the ECM response.

Fibronectin is not only a structural ECM component but also a matrix organizer involved in cell adhesion, matrix assembly, and cell–matrix signaling. Recent work on kidney fibrosis has emphasized that the ECM constitutes a biologically active fibrotic niche capable of regulating cell state through ECM–receptor, integrin, and focal-adhesion signaling (Huang et al., 2023). In mesangial cells, high glucose has also been shown to alter integrin activity and promote Fibronectin matrix assembly (Miller et al., 2014). This classic study was retained because an equally direct recent investigation of high-glucose-induced Fibronectin assembly in mesangial cells was not identified. Consistent with this biological context, the shared disease-associated genes in our study were enriched in ECM–receptor interaction, integrin-related functions, and focal-adhesion pathways. Sample-level pseudobulk analysis further showed higher *Fn1* expression in mesangial cells from DKD mice than in controls, consistent with recent cell-resolved analyses reporting mesangial enrichment of *FN1* in DKD (Lin et al., 2025). This finding does not imply that mesangial cells are the sole renal source of Fibronectin. Instead, it identifies mesangial cells as one relevant compartment in which FN1-associated matrix remodeling may occur.

The association between baicalein and FN1 was generated from CTD- and TCMSP-derived information and should be interpreted according to this evidence level. Such resources integrate chemical, gene, protein, disease, and pharmacological associations collected from heterogeneous sources. Their convergence can prioritize experimentally testable relationships but cannot demonstrate direct molecular recognition. We therefore performed SPR to examine the interaction between baicalein and recombinant human FN1. Baicalein produced a concentration-dependent response, with an apparent *K*D of 8.04 × 10^−5^ M, supporting a direct micromolar-affinity interaction under the tested *in vitro* conditions. However, an interaction measured using immobilized recombinant protein does not establish binding to endogenous FN1, cellular or *in vivo* target engagement, or functional causality. FN1 is therefore more appropriately described as an *in vitro* binding partner and a baicalein-responsive component of ECM remodeling than as a validated cellular or *in vivo* target. This distinction is particularly important in herbal medicine research, where pleiotropic pharmacology requires evidence frameworks that distinguish database-derived associations from experimentally established targets (Ma et al., 2024). Recent experimental studies have shown that baicalein can attenuate Fibronectin accumulation and other fibrotic changes in peritoneal or renal fibrosis models. Related research on baicalin and other *Scutellaria baicalensis* flavonoids has also reported improvement of renal fibrosis in db/db mice, accompanied by regulation of TGF-β/Smad signaling. These studies support the broader antifibrotic pharmacology of *Scutellaria*-derived flavonoids but do not demonstrate that FN1 is a direct target of baicalein.

The animal experiments provide tissue-level evidence that baicalein attenuates renal injury and the profibrotic ECM program. In db/db mice, baicalein treatment improved blood urea nitrogen, serum creatinine, and renal histopathology. Glomerular structural injury, mesangial matrix expansion, and fibrotic deposition were reduced. These changes coincided with lower renal expression of Fibronectin, Collagen I, α-SMA, and TGF-β1 at the transcript or protein level. The agreement between biochemical, molecular, and histological findings strengthens the biological relevance of the observed response. These results are consistent with recent studies reporting antifibrotic effects of baicalein or related *Scutellaria* flavonoids in experimental fibrotic models (Li et al., 2024; Liang et al., 2024). However, the accompanying reduction in blood glucose provides a competing explanation for part of the *in vivo* response. Improvements in renal fibrosis may partly reflect reduced systemic metabolic stress rather than a kidney-specific action on FN1 [6,15]. Nevertheless, baicalein also suppressed fibrosis-associated molecules under a fixed high-glucose condition *in vitro*. Its cellular effects therefore cannot be explained solely by systemic glucose lowering. The metabolic and ECM-related effects of baicalein may contribute in parallel to the overall renal response.

The six-group cellular experiment provides the most direct evidence that FN1 contributes functionally to the observed molecular phenotype. High glucose increased Fibronectin and other fibrosis-associated molecules in mesangial cells, whereas baicalein suppressed this expression pattern. *FN1* overexpression enhanced the profibrotic molecular response and partially weakened the effects of baicalein. Within this cell model, increased *FN1* can therefore further amplify the high-glucose-induced profibrotic molecular phenotype. FN1 represents one functional node through which the cellular response to baicalein is expressed. Importantly, the attenuation produced by *FN1* overexpression was incomplete. This partial rescue is consistent with a non-exclusive contribution of FN1 and with the broader, pleiotropic pharmacology of baicalein. The findings should not be interpreted as complete reversal or evidence that FN1 solely mediates the effects of baicalein.

The relationship between FN1/Fibronectin and TGF-β1 provides a focused direction for future mechanistic investigation. TGF-β signaling is a central regulator of ECM production and fibrotic cell states in DKD and other chronic fibrotic diseases (Massagué and Sheppard, 2023). Fibronectin-rich matrices can influence ECM–receptor signaling and TGF-β-dependent matrix organization (Franco-Valls et al., 2023; Huang et al., 2023). A classic direct study further showed that EDA-Fibronectin enhances the recruitment of latent TGF-β-binding protein-1 to the matrix and influences latent TGF-β activation (Klingberg et al., 2018). In the present study, the parallel changes in FN1/Fibronectin and TGF-β1 raise the possibility of a self-reinforcing interaction between Fibronectin-rich ECM and the TGF-β1-associated profibrotic response. However, this possibility remains a testable hypothesis because the temporal order and reciprocal dependence of FN1 and TGF-β1 were not examined. TGF-β1 should therefore be regarded as a potential downstream component or amplifier of the FN1-associated response rather than as a proven downstream mediator.

Overall, baicalein attenuated DKD-associated renal injury and the profibrotic ECM program. FN1/Fibronectin contributed to this response and could amplify the high-glucose-induced profibrotic molecular phenotype, but did not fully account for the effects of baicalein. The observed relationship between FN1/Fibronectin and TGF-β1 provides a focused hypothesis for reciprocal and pathway-specific experiments rather than a demonstrated positive-feedback mechanism. Accordingly, baicalein is best regarded as a candidate antifibrotic compound whose renal effects may involve both metabolic improvement and modulation of an FN1-associated ECM axis. Further development will require standardized pharmacological characterization, cellular and *in vivo* target-engagement studies, and evidence connecting these molecular responses with clinically relevant renal outcomes (Ren et al., 2025; Poojari et al., 2026).

## 5 Limitations and future directions

This study has several limitations. First, the transcriptomic analyses were retrospective and combined heterogeneous public cohorts, while machine-learning feature selection in modest samples remains susceptible to sampling variability. The model was evaluated in two internal validation cohorts, whereas GSE47184 externally confirmed only the direction of *FN1* expression; the complete marker panel therefore requires prospective external validation. The independently generated single-cell data included only three mice per group. Although each mouse served as the statistical unit, this sample size limits statistical power and sensitivity to rare mesangial cell states. Larger cohorts, spatial transcriptomics, and protein-level validation in human kidney tissues are therefore needed (Jiang et al., 2025a; Jiang et al., 2025b).

Second, the experiments establish functional involvement of FN1 but do not explain how baicalein regulates *FN1* expression or fibronectin accumulation. *FN1* overexpression only partially attenuated the cellular effects of baicalein, and loss-of-function, temporal, and pathway-specific experiments were not performed. The proposed interaction between fibronectin-rich ECM and the TGF-β1-associated response likewise remains a working hypothesis. Reciprocal perturbation of FN1 and TGF-β1, together with measurements of active TGF-β1, phosphorylated FAK, phosphorylated Smad2/3, and assembled fibronectin matrix, will be required to define the directionality of this relationship.

Finally, only one diabetic mouse model was examined, and renal function was assessed using blood urea nitrogen and serum creatinine without albuminuria or filtration-based endpoints. Current DKD standards emphasize albuminuria and glomerular filtration as complementary measures of disease severity and progression (American Diabetes Association Professional Practice Committee for Diabetes, 2026). In addition, the pharmacokinetics, renal exposure, dose–response relationship, long-term efficacy, and safety of baicalein were not systematically evaluated. Future studies should incorporate albuminuria and filtration outcomes, pharmacokinetic and renal-exposure characterization, complementary DKD models, and validation in well-characterized human samples before therapeutic translation.

## Data Availability

Publicly available datasets were analyzed in this study. These datasets are available in the NCBI Gene Expression Omnibus (GEO; https://www.ncbi.nlm.nih.gov/geo/) under accession numbers GSE30528, GSE96804, GSE104948, GSE30529, GSE99339, and GSE47184. The single-cell RNA-sequencing data generated in this study have been deposited in GEO under accession number GSE335999.

## References

[B1] Alicic R. Z. Rooney M. T. Tuttle K. R. (2017). Diabetic kidney disease: challenges, progress, and possibilities. Clin. J. Am. Soc. Nephrol. 12 (12), 2032–2045. 10.2215/cjn.11491116 28522654 PMC5718284

[B2] Amberger J. S. Bocchini C. A. Scott A. F. Hamosh A. (2019). OMIM.org: leveraging knowledge across phenotype-gene relationships. Nucleic Acids Res. 47 (D1), D1038–d1043. 10.1093/nar/gky1151 30445645 PMC6323937

[B3] American Diabetes Association Professional Practice Committee for Diabetes (2026). 11. Chronic kidney disease and risk management: standards of care in diabetes-2026. Diabetes Care 49 (Suppl. 1), S246–s260. 10.2337/dc26-S011 41358881 PMC12690176

[B4] Ashburner M. Ball C. A. Blake J. A. Botstein D. Butler H. Cherry J. M. (2000). Gene ontology: tool for the unification of biology. the gene ontology consortium. Nat. Genet. 25 (1), 25–29. 10.1038/75556 10802651 PMC3037419

[B5] Bakris G. L. Agarwal R. Anker S. D. Pitt B. Ruilope L. M. Rossing P. (2020). Effect of finerenone on chronic kidney disease outcomes in type 2 diabetes. N. Engl. J. Med. 383 (23), 2219–2229. 10.1056/NEJMoa2025845 33264825

[B7] Cox E. Tsuchiya M. T. N. Ciufo S. Torcivia J. Falk R. Anderson W. R. (2025). NCBI taxonomy: enhanced access via NCBI datasets. Nucleic Acids Res. 53 (D1), D1711–d1715. 10.1093/nar/gkae967 39470745 PMC11701650

[B8] Davis A. P. Wiegers T. C. Sciaky D. Barkalow F. Strong M. Wyatt B. (2025). Comparative toxicogenomics Database's 20th anniversary: update 2025. Nucleic Acids Res. 53 (D1), D1328–d1334. 10.1093/nar/gkae883 39385618 PMC11701581

[B9] Dong W. Jiang H. Li Y. Lv L. Gong Y. Li B. (2025). Interpretable machine learning analysis of immunoinflammatory biomarkers for predicting CHD among NAFLD patients. Cardiovasc. Diabetol. 24 (1), 263. 10.1186/s12933-025-02818-1 40611098 PMC12224351

[B10] Dou F. Liu Q. Lv S. Xu Q. Wang X. Liu S. (2023). FN1 and TGFBI are key biomarkers of macrophage immune injury in diabetic kidney disease. Med. Baltim. 102 (45), e35794. 10.1097/md.0000000000035794 37960829 PMC10637504

[B11] Franco-Valls H. Tusquets-Uxó E. Sala L. Val M. Peña R. Iaconcig A. (2023). Formation of an invasion-permissive matrix requires TGFβ/SNAIL1-regulated alternative splicing of fibronectin. Breast Cancer Res. 25 (1), 143. 10.1186/s13058-023-01736-y 37964360 PMC10647173

[B12] Gao Y. Pang X. Zhang H. Li D. Han J. Chen Z. (2025). Immune-metabolic interactions shape the fibrotic landscape of diabetic kidney disease: emerging mechanisms and therapeutic prospects. Front. Physiol. 16, 1736472. 10.3389/fphys.2025.1736472 41648789 PMC12867825

[B13] Hu X. Chen S. Ye S. Chen W. Zhou Y. (2024). New insights into the role of immunity and inflammation in diabetic kidney disease in the omics era. Front. Immunol. 15, 1342837. 10.3389/fimmu.2024.1342837 38487541 PMC10937589

[B14] Huang R. Fu P. Ma L. (2023). Kidney fibrosis: from mechanisms to therapeutic medicines. Signal Transduct. Target Ther. 8 (1), 129. 10.1038/s41392-023-01379-7 36932062 PMC10023808

[B15] Jiang L. Jian J. Zhang H. Wu X. (2025a). Scutellarin attenuated tubule cell apoptosis by modulating HIF-1α for the treatment of DKD: the insight integrating network analysis, machine learning and single-cell transcriptome. Front. Pharmacol. 16, 1656409. 10.3389/fphar.2025.1656409 41089839 PMC12517588

[B16] Jiang L. Yu H. Jian J. Sai X. Wang Y. Zhang Y. (2025b). Landscape analysis of m6A modification regulators reveals LRPPRC as a key modulator in tubule cells for DKD: a multi-omics study. Front. Pharmacol. Volume, 16–2025. 10.3389/fphar.2025.1506896 40255566 PMC12006725

[B17] Klingberg F. Chau G. Walraven M. Boo S. Koehler A. Chow M. L. (2018). The fibronectin ED-A domain enhances recruitment of latent TGF-β-binding protein-1 to the fibroblast matrix. J. Cell Sci. 131 (5), jcs201293. 10.1242/jcs.201293 29361522 PMC5897715

[B18] Korsunsky I. Millard N. Fan J. Slowikowski K. Zhang F. Wei K. (2019). Fast, sensitive and accurate integration of single-cell data with harmony. Nat. Methods 16 (12), 1289–1296. 10.1038/s41592-019-0619-0 31740819 PMC6884693

[B19] Langfelder P. Horvath S. (2008). WGCNA: an R package for weighted correlation network analysis. BMC Bioinforma. 9, 559. 10.1186/1471-2105-9-559 19114008 PMC2631488

[B20] Li J. Zhuang Y. Fan G. Wang S. Yan E. Guo J. (2024). Impact of baicalin and components of Scutellaria baicalensis on renal fibrosis of diabetic kidney disease. Front. Pharmacol. 15, 1480626. 10.3389/fphar.2024.1480626 39712489 PMC11658968

[B22] Li N. Tang T. T. Gu M. Fu Y. Q. Qian W. W. Ma N. N. (2025). Single urinary extracellular vesicle proteomics identifies complement receptor CD35 as a biomarker for sepsis-associated acute kidney injury. Nat. Commun. 16 (1), 6960. 10.1038/s41467-025-62229-4 40730843 PMC12307914

[B23] Li T. Jing H. Gao X. Zhang T. Yao H. Zhang X. (2025). Identification of key genes as diagnostic biomarkers for IBD using bioinformatics and machine learning. J. Transl. Med. 23 (1), 738. 10.1186/s12967-025-06531-1 40611294 PMC12231756

[B24] Liang G. Q. Mu W. Jiang C. B. (2024). Baicalein improves renal interstitial fibrosis by inhibiting the ferroptosis *in vivo* and *in vitro* . Heliyon 10 (7), e28954. 10.1016/j.heliyon.2024.e28954 38601597 PMC11004807

[B25] Lin J. Zheng Y. Mai D. Fang F. Wei Z. Liu M. (2025). Multi-omics and machine learning identify FN1 and ALDH2 as diagnostic biomarkers and therapeutic targets in early and late diabetic kidney disease. Ren. Fail 47 (1), 2577849. 10.1080/0886022x.2025.2577849 41162187 PMC12573553

[B21] Ma L. Feng Z. Y. Li P. (2024). New insights into the use of traditional chinese medicine for treating diabetic kidney disease by regulating DNA methylation. Integr. Med. Nephrol. Androl. 11 (3), e24‐00018. 10.1097/IMNA-D-24-00018

[B26] Massagué J. Sheppard D. (2023). TGF-β signaling in health and disease. Cell 186 (19), 4007–4037. 10.1016/j.cell.2023.07.036 37714133 PMC10772989

[B27] Miller C. G. Pozzi A. Zent R. Schwarzbauer J. E. (2014). Effects of high glucose on integrin activity and fibronectin matrix assembly by mesangial cells. Mol. Biol. Cell 25 (16), 2342–2350. 10.1091/mbc.E14-03-0800 24943838 PMC4142608

[B28] Munjal K. Goel Y. Gauttam V. K. Chopra H. Singla M. Smriti S. (2024). Molecular targets and therapeutic potential of baicalein: a review. Drug Target Insights 18, 30–46. 10.33393/dti.2024.2707 38873988 PMC11168303

[B29] Poojari A. S. Wairkar S. Kulkarni Y. A. (2026). Pharmacokinetics, pharmacodynamics and formulation strategies for enhanced bioavailability of baicalein: an update. Naunyn Schmiedeb. Arch. Pharmacol. 399, 14873–14909. 10.1007/s00210-026-05414-6 42115395

[B6] Ren J. Chang D. Liu J. (2025). Bridging east and west: innovative strategies for herbal medicine development. Integr. Med. Nephrol. Androl. 12 (4). 10.1097/IMNA-D-25-00028

[B30] Ritchie M. E. Phipson B. Wu D. Hu Y. Law C. W. Shi W. (2015). Limma powers differential expression analyses for RNA-sequencing and microarray studies. Nucleic Acids Res. 43 (7), e47. 10.1093/nar/gkv007 25605792 PMC4402510

[B31] Ru J. Li P. Wang J. Zhou W. Li B. Huang C. (2014). TCMSP: a database of systems pharmacology for drug discovery from herbal medicines. J. Cheminform 6, 13. 10.1186/1758-2946-6-13 24735618 PMC4001360

[B32] Sahin K. C. Irmak F. Gultekin M. H. Gokbasi O. Kilic S. Gurses I. (2025). Intralesional baicalein attenuates fibrosis in a rat model of peyronie's disease by inhibiting TGF-β1/smad signaling. Int. J. Impot. Res. 10.1038/s41443-025-01197-1 41115972

[B33] Stelzer G. Rosen N. Plaschkes I. Zimmerman S. Twik M. Fishilevich S. (2016). The genecards suite: from gene data mining to disease genome sequence analyses. Curr. Protoc. Bioinforma. 54, 1.30.1–1.30.33. 10.1002/cpbi.5 27322403

[B34] Stuart T. Butler A. Hoffman P. Hafemeister C. Papalexi E. Mauck W. M. (2019). Comprehensive integration of single-cell data. Cell 177 (7), 1888–1902.e1821. 10.1016/j.cell.2019.05.031 31178118 PMC6687398

[B35] Su S. C. Chang T. C. Chiu Y. W. Chu C. H. Hsu Y. H. Kuo C. S. (2025). 2024 Taiwan clinical practice guideline for diabetic kidney disease - an executive summary. J. Formos. Med. Assoc. 10.1016/j.jfma.2025.08.020 40841287

[B36] Szklarczyk D. Gable A. L. Lyon D. Junge A. Wyder S. Huerta-Cepas J. (2019). STRING v11: protein-protein association networks with increased coverage, supporting functional discovery in genome-wide experimental datasets. Nucleic Acids Res. 47 (D1), D607–d613. 10.1093/nar/gky1131 30476243 PMC6323986

[B37] Tan W. Chen J. Wang Y. Xiang K. Lu X. Han Q. (2024). Single-cell RNA sequencing in diabetic kidney disease: a literature review. Ren. Fail 46 (2), 2387428. 10.1080/0886022x.2024.2387428 39099183 PMC11302482

[B38] Wang Y. Zhang S. Li F. Zhou Y. Zhang Y. Wang Z. (2020). Therapeutic target database 2020: enriched resource for facilitating research and early development of targeted therapeutics. Nucleic Acids Res. 48 (D1), D1031–d1041. 10.1093/nar/gkz981 31691823 PMC7145558

[B39] Yang X. Dou F. Hai L. Xiao Y. Cui J. Cai Y. (2025). Scutellaria baicalensis georgi: a promising source of bioactive molecules for kidney disease therapy. Biomolecules 16 (1), 64. 10.3390/biom16010064 41594603 PMC12838776

[B40] Yang Y. He X. Liu W. Mu L. Wang S. (2025). Comprehensive bioinformatics and *in vivo* validation reveal key molecular drivers of diabetic nephropathy progression. Front. Endocrinol. (Lausanne) 16, 1654401. 10.3389/fendo.2025.1654401 40969366 PMC12440768

[B41] Yu G. Wang L. G. Han Y. He Q. Y. (2012). Cluster profiler: an R package for comparing biological themes among gene clusters. Omics 16 (5), 284–287. 10.1089/omi.2011.0118 22455463 PMC3339379

[B42] Yu Y. Mai Y. Zheng Y. Shi L. (2024). Assessing and mitigating batch effects in large-scale omics studies. Genome Biol. 25 (1), 254. 10.1186/s13059-024-03401-9 39363244 PMC11447944

[B43] Zhang L. Jiang L. Xu R. Zhang X. Zhang B. Yue R. (2025). Epidemiological research on diabetic nephropathy at global, regional, and national levels from 1990 to 2021: an analysis derived from the global burden of disease 2021 study. Front. Endocrinol. (Lausanne) 16, 1647064. 10.3389/fendo.2025.1647064 40937407 PMC12420290

[B44] Zhao B. Liu K. Liu X. Li Q. Li Z. Xi J. (2024). Plant-derived flavonoids are a potential source of drugs for the treatment of liver fibrosis. Phytother. Res. 38 (6), 3122–3145. 10.1002/ptr.8193 38613172

[B45] Zhu R. Lin W. Tang L. Hu Y. (2021). Identification of hub genes associated with adult acute myeloid leukemia progression through weighted gene co-expression network analysis. Aging (Albany NY) 13 (4), 5686–5697. 10.18632/aging.202493 33592582 PMC7950274

