# Supplementary material for: Baicalein attenuates extracellular matrix remodeling via FN1 in diabetic kidney disease: a multi-omics, machine-learning and mesangial-cell rescue study
Source: Front Pharmacol. 2026 Sep 9;17:1852694. doi: 10.3389/fphar.2026.1852694 (PMC13597774; doi:10.3389/fphar.2026.1852694)
Supplement: Supplementary file 1 [file Supplementaryfile1.docx]

**Supplementary Figure S1. Evaluation of batch-effect correction across the three transcriptomic datasets.**
(A, B) Boxplots showing the distributions of gene-expression values across samples from GSE104948, GSE30528 and GSE96804 before (A) and after (B) batch-effect correction using the ComBat method. Colours indicate the source dataset. (C, D) Principal component analysis (PCA) plots showing sample distributions before (C) and after (D) batch-effect correction. Each point represents one biological sample, and colours and shapes indicate the source dataset. The dataset-specific ellipses in (D) delineate the distributions of samples from the three cohorts.

 **Supplementary Figure S2. Selection of the baicalein concentration and treatment duration using the CCK-8 assay.**

(A) Concentration-response analysis of SV40 MES 13 cell viability following treatment with 0, 5, 10, 20, 40, 80, or 160 μM baicalein for 24 h. OD₄₅₀ values were normalized to the mean value of the 0 μM control group, which was defined as 100%. Nonlinear regression yielded an estimated half-maximal inhibitory concentration (IC₅₀) of 34.68 μM. (B) Time-course analysis of OD₄₅₀ values in cells treated with 0 or 20 μM baicalein for 0, 12, 24, or 48 h. Data are presented as the mean ± SD of six technical replicate wells per condition. Based on the concentration-response and time-course results, a sub-IC₅₀ concentration of 20 μM and a treatment duration of 24 h were selected for subsequent cell experiments.

**Supplementary Figure S3. Quality control, dimensionality reduction and cell-type annotation of the single-cell RNA-sequencing dataset.**
(A) Violin plots showing the distributions of the number of detected genes per cell (nFeature_RNA), unique molecular identifier counts per cell (nCount_RNA) and mitochondrial transcript percentage (percent.mt) across the three control and three DKD kidney samples. (B) Principal component analysis plot coloured by biological sample. (C) Dot plot showing the expression of canonical marker genes used to annotate proximal tubular cells (PT), thick ascending limb cells (TAL), collecting duct principal cells (CD-PC), collecting duct intercalated cells (CD-IC), podocytes, endothelial cells (EC), mesangial cells (MC), B cells, T cells, natural killer cells (NK cells), monocytes and granulocytes. Dot size represents the percentage of cells expressing each gene, and colour intensity represents the average expression level. (D) t-distributed stochastic neighbour embedding (t-SNE) plot showing the 12 annotated kidney cell populations. Each point represents one cell, and colours indicate cell identity. (E) Relative proportions of the annotated cell populations in the control and DKD groups. DKD, diabetic kidney disease.

**Supplementary Figures S4 and S5. Uncropped western blot images of renal extracellular matrix and fibrosis-associated proteins in DKD mice.**

(A–D) Uncropped western blot images of collagen I (A), fibronectin (B), TGF-β1 (C), and α-smooth muscle actin (α-SMA; D), together with the corresponding GAPDH loading controls. Each supplementary figure includes three independent biological replicates per group, and the two figures together represent six mice per group. In each figure, lanes 1–3 represent the Control group, lanes 4–6 represent the DKD group, and lanes 7–9 represent the DKD + BAI group. Molecular-weight markers are shown to the left of each membrane. These images correspond to the representative western blots and densitometric analyses presented in Fig. 7E–H, which included six biological replicates per group. BAI, baicalein; DKD, diabetic kidney disease.

**Supplementary Figure S6. Uncropped western blot images from the FN1-overexpression rescue experiment in high-glucose-treated mesangial cells.**
(A–D) Uncropped western blot images of α-SMA (A), collagen I (B), fibronectin (C) and TGF-β1 (D), together with their corresponding α-tubulin loading controls. Three independent experiments are shown for each protein. Within each blot, lanes from left to right represent NG + Vector, MA + Vector, HG + Vector, HG + BAI + Vector, HG + BAI + FN1-OE and HG + FN1-OE. Molecular-weight markers are shown to the left of each membrane. These images correspond to the representative western blots and densitometric analyses presented in Fig. 9E–H. BAI, baicalein; FN1-OE, FN1 overexpression; HG, high glucose; MA, mannitol osmotic control; NG, normal glucose.

**Supplementary Table S1. Primer sequences used for RT-qPCR analyses**

| **Gene** | **Forward primer (5′–3′)** | **Reverse primer (5′–3′)** | **Application** |
| --- | --- | --- | --- |
| *Gapdh* | AGGTCGGTGTGAACGGATTTG | GGGGTCGTTGATGGCAACA | Mouse kidney tissues |
| *Tuba1a* | TATGCCTGCTGGGAGCTCTA | AAGGAGTCATCTCCTCCCCC | Mesangial cells |
| *Acta2* | GGGAGTAATGGTTGGAATG | TCGGATACTTCAGCGTCA | Mouse kidney tissues and mesangial cells |
| *Fn1* | TCAATCCAAATGCCTCTAC | GAACCTTGTGCCTCCTCT | Mouse kidney tissues and mesangial cells |
| *Col1a1* | CGCCATCAAGGTCTACTGC | GAATCCATCGGTCATGCTCT | Mouse kidney tissues and mesangial cells |
| *Tgfb1* | ACTGGAGTTGTACGGCAGTG | GGGGCTGATCCCGTTGATTT | Mouse kidney tissues and mesangial cells |

**Note:** Primer sequences are presented in the 5′–3′ orientation. *Gapdh* and *Tuba1a* were used as the reference genes for the mouse kidney tissue and mesangial-cell experiments, respectively. The Acta2, Fn1, Col1a1 and Tgfb1 primer pairs were used in both experimental settings. RT-qPCR, reverse-transcription quantitative polymerase chain reaction.
